# Supplementary material for: Quality by design for mRNA platform purification based on continuous oligo-dT chromatography
Source: Mol Ther Nucleic Acids. 2024 Sep 11;35(4):102333. doi: 10.1016/j.omtn.2024.102333 (PMC11458983; doi:10.1016/j.omtn.2024.102333)
Supplement: Document S1. Figures S1‒S4 and Tables S1‒S12 [file mmc1.pdf]

OMTN, Volume *35*

Supplemental information

Quality by design for mRNA platform purification based on continuous oligo-dT chromatography

Jixin Qu, Adithya Nair, George W. Muir, Kate A. Loveday, Zidi Yang, Ehsan Nourafkan, Emma N. Welbourne, Mabrouka Maamra, Mark J. Dickman, and Zoltán Kis

ANOVA analysis

The three-factor at three-level Box-Behnken factorial design was applied for the ANOVA analysis with the following equation, where X is the independent variable chosen by the experimental design, Y is the process response (dependent variable), k is the number of patterns, i and j are index number of patterns, b_0 is the intercept term, b_i is the linear main effect, b_{ii} is the quadratic effect, b_{ij} is the interaction effect and ε is the random error [31].

S1 $$Y = b_0 + \sum_{i=1}^{k} b_i X_i + \sum_{i=1}^{k} b_{ii} X_i^2 + \sum_{i=1}^{k} \sum_{j>1}^{k} b_{ij} X_i X_j + \varepsilon$$

Productivity calculations

Productivity is defined as the amount of mRNA that can be purified per unit time (e.g. minute) per unit scale of the process (e.g. mL of the chromatography resin). The productivity difference between batch and continuous chromatography was calculated based on two different methods:

1) based on one single run;
2) based on running the chromatography unit operation non-stop for 24 hours.

For the productivity of one single run, the amount of mRNA purified by batch and continuous chromatography per run was 4.26 and 18.47 mg, respectively. The time consumed, excluding start-up equilibration and shut-down clean-in-place (CIP), was 14 minutes for batch and 20 minutes for continuous chromatography. The chromatography resin applied is 1 mL for both (for continuous chromatography, only 1 mL of the resin was fully utilised at a time). Thus, the productivity was calculated as 0.30 mg/min/mL for the batch and 0.92 mg/min/mL for the continuous chromatography unit operations. Therefore, based on the productivity of one single run, continuous chromatography is 3-fold more productive than the batch chromatography unit operation.

For the productivity of running the chromatography system for 24 hours, 5 minutes of preparation time was included for both batch and continuous between every two runs and the time consumed in total, 22 minutes for batch and 25 minutes for continuous, were taken into account, including start-up equilibration and shut-down CIP. For batch chromatography, there was a 5-minute preparation time between consecutive runs, 4.26 mg of mRNA could be purified each cycle, and 1 mL chromatography resin was applied. It was concluded that 53.33 cycles can be conducted in 24 hours, and this has given a productivity of 0.16 mg/min/mL. For continuous chromatography, only one start-up equilibration (5 minutes) was applied in the initial cycle, and 5 minutes of preparation time was included at the beginning, then the system ran non-stop for 24 hours. Each cycle purified 18.47 mg of mRNA, and this concluded with

71.5 cycles (4 bind-elute stages per cycle) to be conducted in 24 hours and given 0.92 mg/min/mL of productivity. Therefore, based on the 24-hour productivity calculation, continuous chromatography is 5.75-fold more productive than the batch chromatography unit operation. These productivity differences are expected to be maintained under Good Manufacturing Practice (GMP) compliant manufacturing conditions as well, where column volumes might be larger, and more time is required to set up the process (e.g. due to documentation, verification and compliance requirements).

Cost calculations

The cost calculation details are available below in Supplementary Table 10. The operating cost distribution for chromatography unit operations in a GMP facility was estimated based on techno-economic modelling results for the mRNA manufacturing process in a GMP facility, and the following distribution of the cost components was assumed [1,2]:

A) raw materials costs 60% of total costs;

B) chromatography resin costs: 30% of total costs;

C) labour costs: 4.5% of total costs;

D) facility and other fixed costs: 5.5% of total costs.

The cost calculation for the raw materials is based on the percentage mRNA yield of the batch and continuous chromatography unit operations, 85% and 92.6%, respectively. This 7.6% (92.6% minus 85%) yield difference accounts for 60% of the total cost savings. Therefore, a 4.5% (7.6% multiplied by 60%) total cost reduction for raw materials is achievable when transitioning from batch to continuous chromatography.

The cost of the chromatography resin is calculated based on the amount of mRNA that can be purified in batch and continuous, using 1 mL of the chromatography resin for bind-elute cycle, 4.26 mg and 4.62 mg, respectively. These numbers were converted from mg to percentage values. Therefore, there is an 8.45% difference between batch and continuous chromatography. The chromatography resin costs account for 30% of the total costs. Therefore, 2.34% (8.45% multiplied by 30%) of the total costs are reduced due to chromatography resin savings when transitioning from batch to continuous chromatography.

The labour cost difference between batch and continuous chromatography was calculated based on the difference in the productivity between batch and continuous chromatography unit operations. The continuous chromatography was more productive than the batch chromatography by a factor of 5.75 over a period of 24 hours. It was assumed that the labour cost in batch chromatography was 100%, which was reduced by the 5.75 productivity factor of

17.39% in the case of continuous chromatography, resulting in a labour cost reduction of 82.61%. Labour costs only account for 4.5% of the total GMP costs of the chromatography unit operation. Therefore, the total labour cost reduction of continuous chromatography compared to batch is 82.61% multiplied by 4.5% equals 3.72%.
The facility and other fixed costs difference between batch and continuous chromatography were calculated based on the difference in the productivity between batch and continuous chromatography unit operations. The continuous chromatography was more productive than the batch chromatography by a factor of 5.75 over a period of 24 hours. It was assumed that the facility and other fixed costs in batch chromatography were 100%, and this was reduced by the 5.75 productivity factor to 17.39% in the case of continuous chromatography, resulting in a facility and other fixed costs reduction of 82.61%. Facility and other fixed costs only account for 5.5% of the total GMP costs of the chromatography unit operation. Therefore, the total labour cost reduction of continuous chromatography compared to batch is 82.61% multiplied by 5.5% equals 4.54%.
The total operating cost reduction when operating a continuous process compared to a batch process is calculated by summing up the cost reduction of all the cost components: 4.56% plus 2.54% plus 3.72 plus 4.54 equals 15.36%.

Cell transfection results

The cell transfection work was performed with human embryonic kidney 293 cells (HEK 293) obtained from the American Type Culture Collection (ATCC, VA, USA). Cells were seeded at a concentration of 3 x 10^5 cells/mL in the medium of FluoroBrite DMEM (Thermo Fisher Scientific, MA, USA), supplemented with 10% fetal bovine serum (FBS, Sigma Aldrich, MO, USA) and 1% penicillin-streptomycin (Gibco, MA, USA). mRNA transfection was performed using the TransIT-mRNA Transfection Kit (Mirusbio, WI, USA) according to the manufacturer's instructions. As shown in Figure S1, 6 different conditions with the same loading concentration of 1000 ng/µL, mRNA purified by batch chromatography and continuous chromatography were transfected in cells, together with crude IVT mRNA, mRNA purified by Monarch purification kit (New England Biolabs, MA, USA), DNA template and cell only as comparison. The detection of the transfected cells was performed using a SpectraMax iD 5 Multi-Mode Microplate Reader (Molecular Devices, CA, USA). Fluorescence was measured with an excitation wavelength of 482 nm and an emission wavelength of 535 nm. The experiment was repeated three times independently; consistent

results were observed across all replications, demonstrating the reliability of the assay conditions.

Table S1. The errors of UV-spectroscopy, Capillary Gel Electrophoresis (CGE) and Anion Exchange High-Performance Liquid Chromatography (AEX HPLC) analytical methods.

Analytical method	Relative standard deviation [%]	Limit of detection [ng]
UV-spectroscopy	2	2
CGE	1.2	1
AEX-HPLC	4	8 for eGFP mRNA detection
		26 for eGFP mRNA quantification

Table S2. Uncertainty and impact score for the risk assessment of the continuous chromatography process, adopted from Simon et al. (2022). [3]

Uncertainty score		Rationale
5	Very high	No information found
4	High	Limited supportive information
3	Moderate	Product understanding
2	Low	Specific non-clinic studies or clinic studies of similar technologies
1	Very low	Clinical studies

Impact score	Rationale
2	No impact
8	Moderate impact
25	Major impact

Table S3. QA assessment criteria for the criticality of the continuous chromatography QAs, the framework was adopted from Simon et al. (2022). [3]

QA
QA/CQA
pCQA
CQA

Impact \ Uncertainty	1	2	3	4	5
2	2	4	6	8	10
8	8	16	24	32	40
25	25	50	75	100	125

Table S4. Risk assessment of quality attributes for the chromatography process, modified from Simon et al. (2022). [3]

CQA	Impact Score - Safety	Impact Score Efficacy	Uncertainty Score - Safety	Uncertainty Score - Efficacy	Severity Score - Safety	Severity Score - Efficacy	Max Severity	Classification	References
**RNA content**	2	8	3	3	8	24	24	CQA*	4
**RNA sequence identity**	8	25	4	3	32	75	75	CQA	4
**RNA structure integrity**	8	25	4	3	32	75	75	CQA	4
**5' capping efficiency**	2	25	3	2	6	50	50	CQA	5–7
**PolyA tail length**	2	25	4	3	8	75	75	CQA	6,8
**PolyA tail level**	8	25	4	2	32	50	50	CQA	6,8
**RNA purity**	25	25	2	3	50	75	75	CQA	9
**dsRNA species**	25	25	2	3	50	75	75	CQA	10,11
**Shorter RNA species**	25	25	3	3	75	75	75	CQA	12
**RNA precipitates**	2	8	4	4	8	32	32	pCQA	4
**Residual DNA template**	8	8	3	3	24	24	24	QA	13
**Residual host cell proteins**	8	8	4	4	32	32	32	pCQA	14,15
**Residual enzymes**	8	25	4	3	32	75	75	pCQA	16,17
**Residual impurities**	8	8	3	4	24	32	32	pCQA	18,19
**Residual solvents**	8	8	3	4	24	32	32	pCQA	20
**pH**	2	25	3	3	6	75	75	CQA	12,21
**Appearance**	2	2	3	3	6	6	6	QA	22
**Viscosity**	2	2	4	3	8	6	8	QA	23
**Immunogenicity**	8	25	2	3	50	24	50	CQA	5,24–27
**Potency / In Vitro Expression**	2	25	2	2	50	4	50	CQA	12,28–30

Table S5. Assessment criteria for evaluating the uncertainty and impact of the CPPs and CMAs on product CQAs and manufacturing KPIs.

Uncertainty assessment		Impact assessment	
Uncertainty rating	Uncertainty rating rationale	Impact score*	Descriptor
High^4	Limited supportive information about the relation.	0	Negligible
Moderate^3	Process expert knowledge only, or information taken from other similar processes.	+, -, ±,∓1	Minor
Low^2	Relation is supported by both expert knowledge and the literature on the same or very similar processes.	+, -, ±,∓2	Moderate
Very Low^1	Relation is supported by a large number and consistent published data or results, information specific to the mRNA process.	+, -, ±,∓3	Major

* The “^4 to ^1” in superscript in the table indicates the significance of uncertainty, from very high uncertainty “^1” to very low uncertainty “^1”. The “+” indicates a positive linear slope, the “-” sign indicates a negative linear slope, and the “±” indicates a peak trend (initial increase, reaching a maximum followed by a decrease) when plotting CQAs or KPIs as a function of PPs or CMAs. The “∓” indicates a valley trend (opposite, inverse of the peak trend), an initial decrease, reaching a minimum followed by an increase when plotting CQAs or KPIs as a function of PPs or CMAs.

Table S6. Definitions of Key Performance Indicators (KPIs)

**KPI**	**Definition**
mRNA yield [mg]	The amount of mRNA recovered after the purification by chromatography
Eluted mRNA amount [mg]	The amount of mRNA eluted in the elution phase

Process time [min]	The processing time used for the chromatography process, excluding the time for the start-up equilibration and shut-down phases
Column switch interval [min]	The time measurement between the column switches, as the column switch is dictated by the Breakthrough UV absorbance at 280 nm
Phase time for continuity [min]	The time measurement for each phase time, as the consideration for the continuity of the chromatography process
Productivity [mg/min/mL]	The amount of mRNA purified per minute per mL of the chromatography resin

Table S7. The independent and dependent variables of the experimental optimisation.

Independent variable	Low level (-1)	Medium level (0)	High level (+1)	Dependent variable
X1: guanidine-HCl concentration (M)	0.3	0.6	0.9	Y1 = yield (mg)
X2: mRNA load concentration (mg/mL)	0.3	0.5	0.7	Y2 = mRNA integrity (%)
X3: Load flow rate (mL/min)	1	3	5	Y3 = purity (%)

Table S8. Output of ANOVA analysis.

$Yield=70.304-3.84\times X1-15.107\times X2+7.194\times X3-2.0562\times X1^2+7.9088\times X2^2$
$14.256\times X3^2+1.75\times X1X2+7.535\times X1X3+0.8925\times X2X3$

Factor	Sum of squares	DF	Mean of squares	F	P Value
X1	147.46	1	147.46	2.346	0.17649
X2	2282.2	1	2282.2	36.31	0.00094307
X3	517.54	1	517.54	8.234	0.028446
$X1^2$	11.147	1	11.147	0.17734	0.68834
$X2^2$	164.9	1	164.9	2.6236	0.15641
$X3^2$	535.81	1	535.81	8.5248	0.026641
X1X2	24.5	1	24.5	0.3898	0.55593
X1X3	454.21	1	454.21	7.2265	0.096139
X2X3	6.3724	1	6.3724	0.10139	0.76096
Residual error	377.12	6	52.854		
Total	4473	15	298.2		

Table S9. The independent and dependent variables of the experimental optimisation

Independent variable	Low level (-1)	Medium level (0)	High level (+1)	Dependent variable
X1: mRNA load concentration (mg/mL)	0.1	0.25	0.4	Y1 = yield (mg)
X2: Load flow rate (mL/min)	2	4	6	Y2 = mRNA integrity (%)<br>Y3 = purity (%)

Table S10. Output of ANOVA analysis, Yield=a-b×X1-c×X2+d× $X1^2$+e×$X2^2$-f×X1X2

Factor	Sum of squares	DF	Mean of squares	F	P Value
X1	0.081448	1	0.081448	92.783	0.002375
X2	0.0029007	1	0.0029007	3.3044	0.16669
$X1^2$	0.018348	1	0.018348	20.902	0.019635
$X2^2$	0.0022916	1	0.0022916	2.6106	0.20457
X1X2	0.011618	1	0.011618	13.235	0.035792
Residual error	0.0026335	3	0.00087784		
Total	0.11924	8	0.014905		

Table S11. Details of operating cost calculations for batch vs continuous chromatography.

	**Batch Chromatography**	**Continuous Chromatography**
Amount of mRNA purified each cycle [mg][(A)]	4.26	18.47
Time consumed excluding start-up and shut-down [min][(B)]	14	20
Time consumed in total [min][(C)]	22	25
Chromatography resin applied [mL][(D)]	1	1
Preparation time [min][(E)]	5	5
Productivity [mg/min/mL] for one run[(F)]	0.30	0.92
Productivity [mg/min/mL] of running for 24 hours[(G)]	0.16	0.92

Productivity increase for a single run: 3.07 fold
Productivity increase of running for 24 hours: 5.75 fold

(A) The amount of mRNA recovered from the chromatography process, quantified by AEX HPLC.
(B) The time consumed for load, wash and elution phases, excluding start-up equilibration and shut-down column CIP.
(C) The total process time including start-up equilibration, load, wash, elution and shut-down column CIP.

(D) The chromatography resin that is fully utilised. For continuous chromatography, only 1 mL resin is fully utilised at a time, as the second column in the loading zone is only catching the flowthrough mRNA from the first column.
(E) The time consumed for preparing buffers, samples equipment and consumables.
(F) The productivity calculated for a single run, considering the amount of mRNA purified each cycle, time consumed excluding start-up and shut-down and chromatography resin applied.
(G) The productivity calculated for running the chromatography process for 24 hours non-stop, considering the amount of mRNA purified each cycle, time consumed in total, chromatography resin applied and preparation time.

Table S12. Details of operating cost calculations for batch vs continuous chromatography.

	**Batch Chromatography**	**Continuous Chromatography**	**Difference [%]**	**Proportion of total costs [%]***	**Cost reduction [%]**
Raw materials costs [%] (A)	85	92.6	7.6	60	4.56
Chromatography resin costs [%] (B)	4.26	4.62	8.45	30	2.54
Labour costs [%] (C)	100	17.39	82.61	4.5	3.72
Facility and other costs [%] (D)	100	17.39	82.61	5.5	4.54
				**Total cost reduction:**	**15.36 %**

* The proportion of total operating costs was calculated based on techno-economic modelling results[1,2].
(A) The raw materials cost difference was calculated based on yield differences between batch (85%) and continuous (92.6%) chromatography.
(B) The chromatography resin cost difference was calculated based on the difference in the amount of mRNA purified per mL of resin between batch and continuous chromatography.
(C) The labour cost difference was calculated based on the difference in the productivity between batch and continuous, assuming batch at 100% cost and continuous was reduced by the productivity factor difference (of 5.75) between batch and continuous.
(D) The facility and other costs difference was calculated based on the difference in the productivity between batch and continuous, assuming batch at 100% cost and continuous was reduced by the productivity factor difference (of 5.75) between batch and continuous.

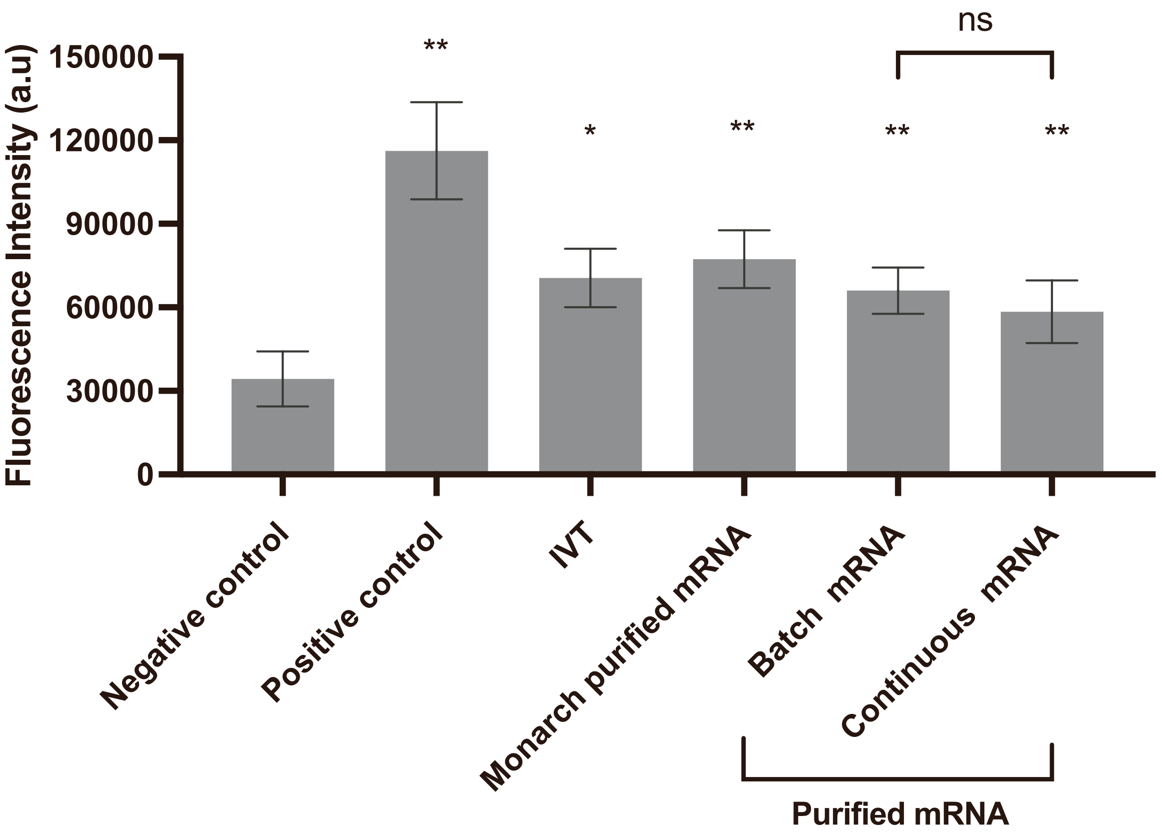

Figure S1. Fluorescence measurements of various mRNA samples using the Spectra Max 24-well plate reader (Excitation: 485 nm, Emission: 535 nm). The samples tested include negative control (cell only), positive control (plasmid DNA), IVT, Monarch kit purified mRNA, mRNA purified by batch chromatography and mRNA purified by continuous chromatography. The results demonstrate the fluorescence intensity of each sample after 24 hours of incubation, indicating the relative expression levels. Statistical significance is indicated as follows: *, $p<0.05$ (significant), **, $p<0.01$ (very significant) and ns (not significant) compared to the negative control.

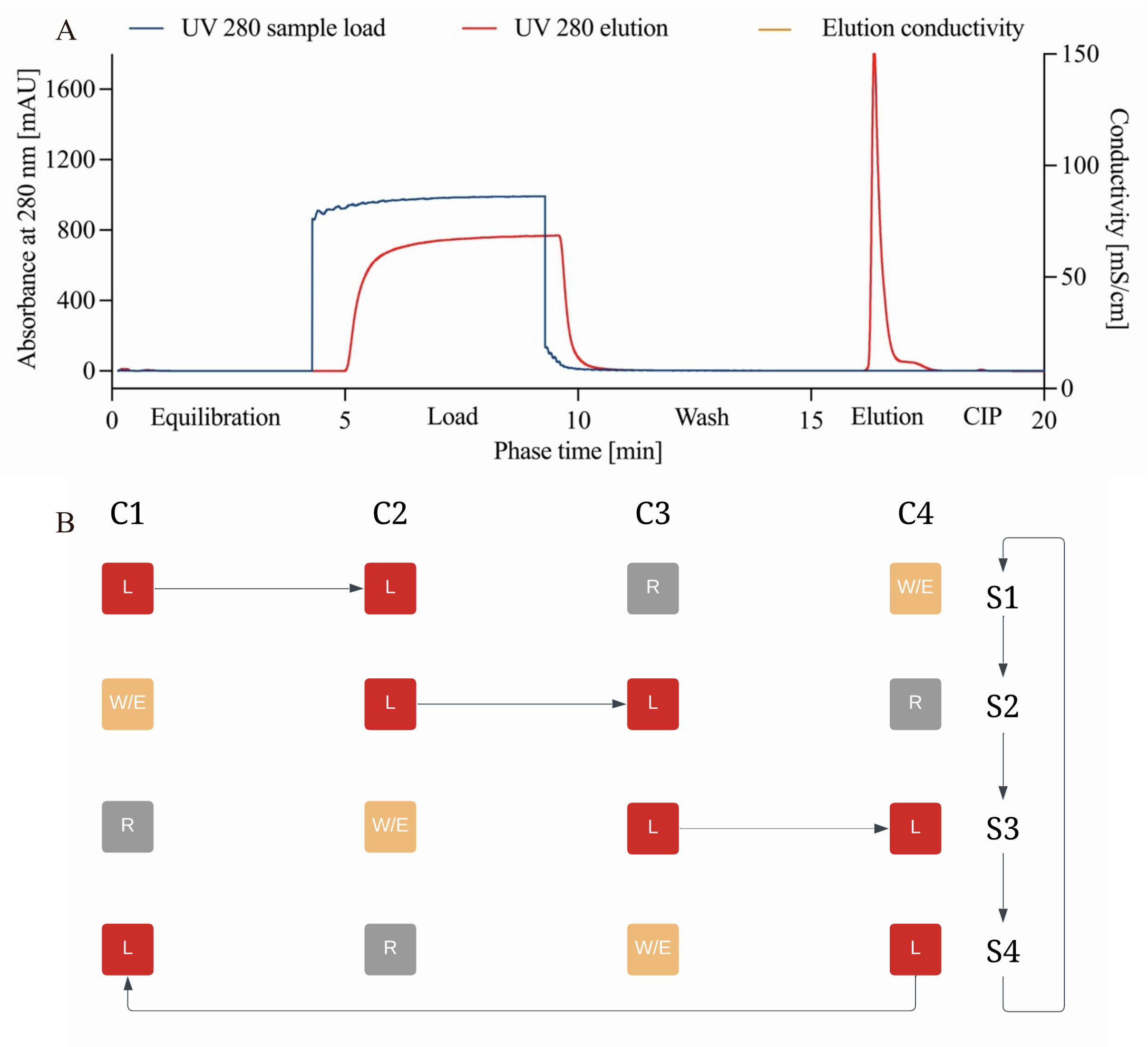

Figure S2. Oligo-dT batch chromatogram and illustration of the continuous chromatography process. **A**: One column in-batch oligo-dT chromatography process. **B.** Schematic of four-column periodic counter-current (PCC) continuous chromatography. C1-C4: columns 1-4; L: Load; W: wash; R: regeneration; E: elution and S: switch.

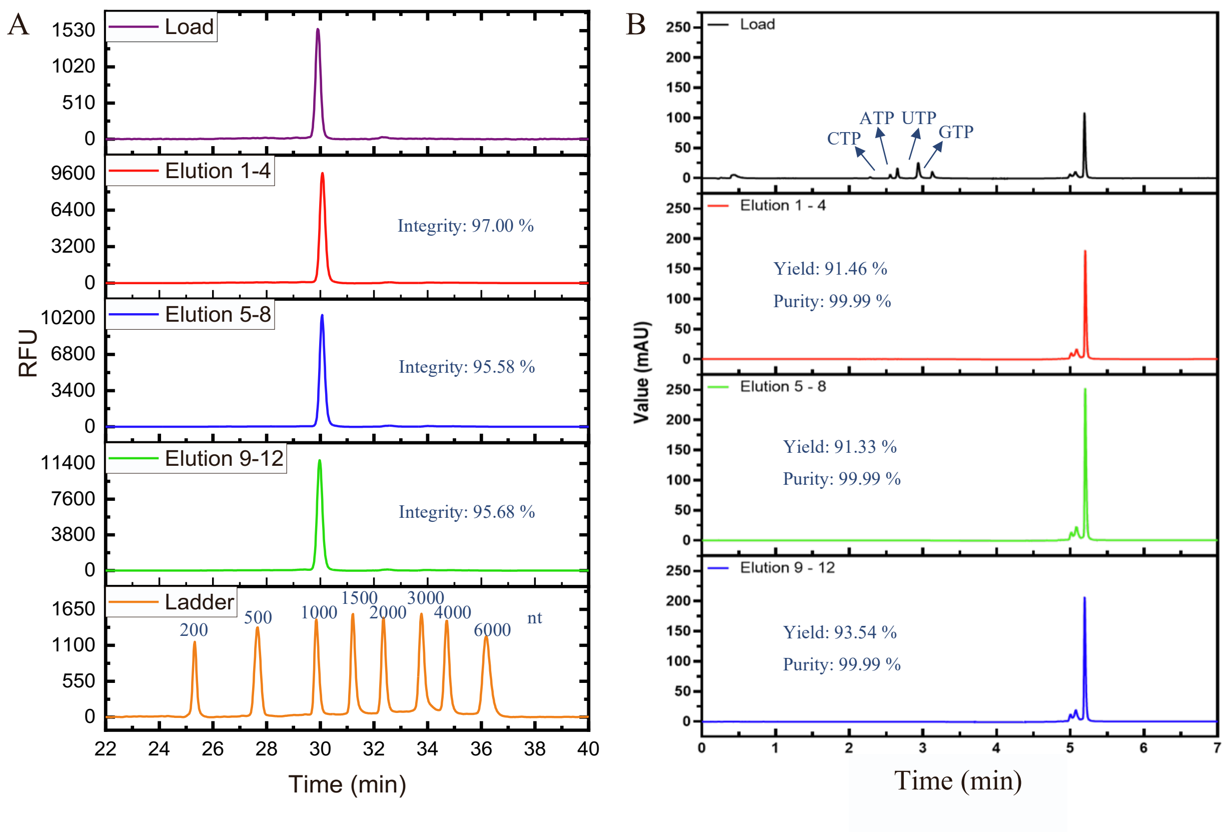

Figure S3. A representative example of CGE and HPLC analysis results. **A**. Examples of CGE analysis of mRNA integrity. mRNA fragments are separated and detected by fluorescence based on their sizes and the integrity of RNA is determined by comparing with the mRNA ladder. Examples of AEX HPLC analysis of mRNA yield and purity. AEX HPLC separates the molecules based on their charge and quantifies the amount of mRNA and NTPs using UV absorbance at the wavelength of 260 nm.

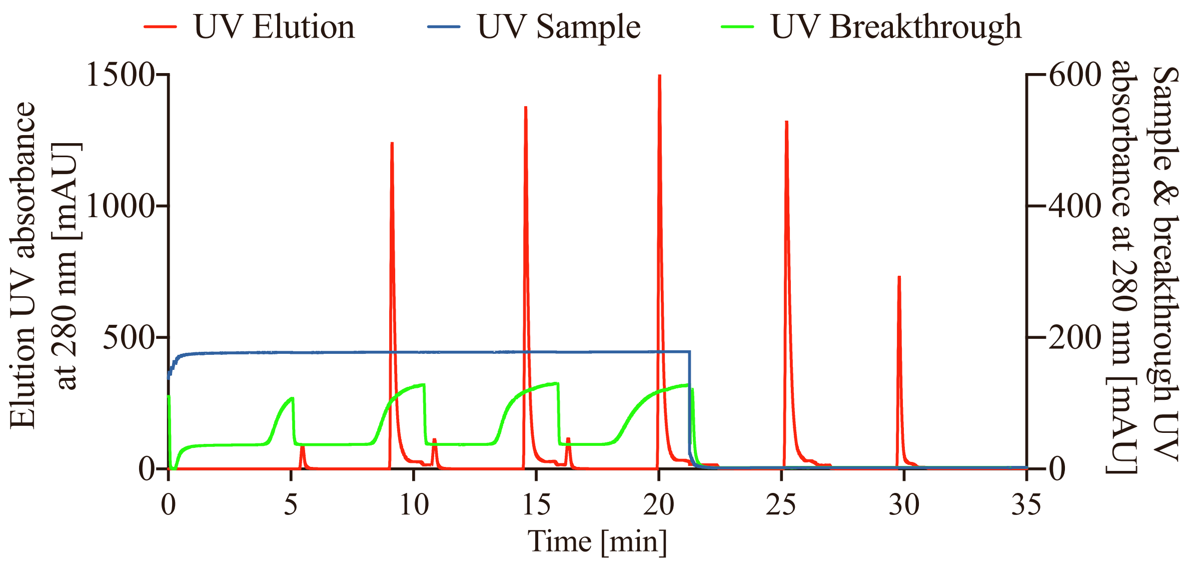

Figure S4. Chromatogram of the continuous oligo-dT purification of the 4284 nucleotides long SARS-CoV-2 Spike Protein encoding mRNA. The total salt concentration was 600 mM guanidine-HCl, the SARS-CoV-2 Spike Protein mRNA load concentration was 0.1 mg/mL, the load volume was 20 mL for each load-elute loop and the load flow rate was 4 mL/min. In total, 1 cycle of 4 load-elute loops was performed using four 1 mL monolith oligo-dT columns under the same conditions as the test performed with eGFP encoding mRNA. The total run time was 32 minutes, excluding the start-up equilibration and shut-down phases. In total, 7.56 mg of purified mRNA was eluted out of the 8 mg unpurified crude IVT mRNA loaded, giving a yield of 94.5%. The purified mRNA integrity was >90% and the purity was 99.9%.

References

1. Kis, Z., Kontoravdi, C., Shattock, R., and Shah, N. (2020). Resources, Production Scales and Time Required for Producing RNA Vaccines for the Global Pandemic Demand. Vaccines 2021, Vol. 9, Page 3 *9*, 3. 10.3390/VACCINES9010003.
2. Kis, Z., Tak, K., Ibrahim, D., Papathanasiou, M.M., Chachuat, B., Shah, N., and Kontoravdi, C. (2022). Pandemic-response adenoviral vector and RNA vaccine manufacturing. npj Vaccines 2022 7:1 *7*, 1–10. 10.1038/s41541-022-00447-3.
3. Daniel, S., Kis, Z., Kontoravdi, C., and Shah, N. (2022). Quality by Design for enabling RNA platform production processes. Preprint at Elsevier Ltd, 10.1016/j.tibtech.2022.03.012 10.1016/j.tibtech.2022.03.012.
4. van de Berg, D., Kis, Z., Behmer, C.F., Samnuan, K., Blakney, A.K., Kontoravdi, C., Shattock, R., and Shah, N. (2021). Quality by design modelling to support rapid RNA vaccine production against emerging infectious diseases. NPJ Vaccines *6*. 10.1038/s41541-021-00322-7.
5. Evaluation of the quality, safety and efficacy of messenger RNA vaccines for the prevention of infectious diseases: regulatory considerations (2021).
6. Chang Kim, S., Singh Sekhon, S., Shin, W.-R., Ahn, G., Cho, B.-K., Ahn, J.-Y., and Kim, Y.-H. (2022). Modifications of mRNA vaccine structural elements for improving mRNA stability and translation efficiency. *18*, 1–8. 10.1007/s13273-021-00171-4.
7. Decroly, E., Ferron, F., Lescar, J., and Canard, B. (2011). Pre-mRNA splicing Conventional and unconventional mechanisms for capping viral mRNA. Nat Rev Microbiol *10*, 51. 10.1038/nrmicro2675.
8. Bernstein, P., and Ross, J. (1989). Quantum Mechanical Tunneling in Biological Systems.) Acc. ('hem. Res *14*, 13650–13662.

9. Vermeulen, J., De Preter, K., Lefever, S., Nuytens, J., De Vloed, F., Derveaux, S., Hellemans, J., Speleman, F., and Vandesompele, J. (2011). Measurable impact of RNA quality on gene expression results from quantitative PCR. Nucleic Acids Res *39*. 10.1093/nar/gkr065.
10. Foster, J.B., Choudhari, N., Perazzelli, J., Storm, J., Hofmann, T.J., Jain, P., Storm, P.B., Pardi, N., Weissman, D., Waanders, A.J., et al. Purification of mRNA Encoding Chimeric Antigen Receptor Is Critical for Generation of a Robust T-Cell Response. 10.1089/hum.2018.145.
11. Karikó, K., Muramatsu, H., Ludwig, J., and Weissman, D. (2011). Generating the optimal mRNA for therapy: HPLC purification eliminates immune activation and improves translation of nucleoside-modified, protein-encoding mRNA. Nucleic Acids Res *39*, e142–e142. 10.1093/NAR/GKR695.
12. CHMP (2021). COVID-19 Vaccine Moderna, INN-COVID-19 mRNA Vaccine (nucleoside modified).
13. Wang, Y., Cooper, R., Kiladjian, A., Bergelson, S., and Feschenko, M. (2019). A Digestion-free Method for Quantification of Residual Host Cell DNA in rAAV Gene Therapy Products. Mol Ther Methods Clin Dev *13*, 526–531. 10.1016/j.omtm.2019.05.005.
14. Pharmacopeia, U.S. (2016). Residual Host Cell Protein Measurement in Biopharmaceuticals USP 39 NF 34 THE NATIONAL FORMULARY á1132ñ Residual Host Cell Protein Measurement in Biopharmaceuticals.
15. Singh Rathore, A., Sobacke, S.E., Kocot, T.J., Morgan, D.R., Dufield, R.L., and Mozier, N.M. (2003). Analysis for residual host cell proteins and DNA in process streams of a recombinant protein product expressed in Escherichia coli cells. J Pharm Biomed Anal *32*, 1199–1211. 10.1016/S0731-7085(03)00157-2.
16. Maruggi, G., Zhang, C., Li, J., Ulmer, J.B., and Yu, D. (2019). mRNA as a Transformative Technology for Vaccine Development to Control Infectious Diseases. Molecular Therapy *27*. 10.1016/j.ymthe.2019.01.020.
17. Tagliabue, A., Stadler, K., Rauch, S., Jasny, E., Schmidt, K.E., and Petsch, B. (2018). New Vaccine Technologies to Combat Outbreak Situations. Frontiers in Immunology | www.frontiersin.org *9*. 10.3389/fimmu.2018.01963.
18. Mathieu, E., Ritchie, H., Ortiz-Ospina, E., Roser, M., Hasell, J., Appel, C., Giattino, C., and Rodés-Guirao, L. (2020). Coronavirus Pandemic (COVID-19). Our World in Data *5*, 947–953. 10.1038/S41562-021-01122-8.

19. Lanigan, R.S., Yamarik, T.A., and Andersen, F.A. (2002). Final report on the safety assessment of EDTA, calcium disodium EDTA, diammonium EDTA, dipotassium EDTA, disodium EDTA, TEA-EDTA, tetrasodium EDTA, tripotassium EDTA, trisodium EDTA, HEDTA, and trisodium HEDTA. Int J Toxicol *21 Suppl 2*, 95–142. 10.1080/10915810290096522.
20. Step ICH guideline Q3C (R8) on impurities: guideline for residual solvents.
21. van der Helm, M.W., van der Meer, A.D., Eijkel, J.C.T., van den Berg, A., and Segerink, L.I. (2016). Microfluidic organ-on-chip technology for blood-brain barrier research. https://doi.org/10.1080/21688370.2016.1142493 *4*. 10.1080/21688370.2016.1142493.
22. Poveda, C., Biter, A.B., Bottazzi, M.E., Strych, U., Biter@bcm, A., and Edu, A.B.B. Establishing Preferred Product Characterization for the Evaluation of RNA Vaccine Antigens. 10.3390/vaccines7040131.
23. EUROPEAN PHARMACOPOEIA.
24. CHMP Committee for Medicinal Products for Human Use (CHMP) Assessment report Comirnaty Common name: COVID-19 mRNA vaccine (nucleoside-modified).
25. Vlatkovic, I. (2021). Non-Immunotherapy Application of LNP-mRNA: Maximizing Efficacy and Safety. Biomedicines 2021, Vol. 9, Page 530 *9*, 530. 10.3390/BIOMEDICINES9050530.
26. Kalnin, K. V, Plitnik, T., Kishko, M., Zhang, J., Zhang, D., Beauvais, A., Anosova, N.G., Tibbitts, T., DiNapoli, J., Ulinski, G., et al. ARTICLE Immunogenicity and efficacy of mRNA COVID-19 vaccine MRT5500 in preclinical animal models. 10.1038/s41541-021-00324-5.
27. Heine, A., Juranek, S., and Brossart, P. (2021). Clinical and immunological effects of mRNA vaccines in malignant diseases. Preprint at BioMed Central Ltd, 10.1186/s12943-021-01339-1 10.1186/s12943-021-01339-1.
28. Corbett, K.S., Flynn, B., Foulds, K.E., Francica, J.R., Boyoglu-Barnum, S., Werner, A.P., Flach, B., O'Connell, S., Bock, K.W., Minai, M., et al. (2020). Evaluation of the mRNA-1273 Vaccine against SARS-CoV-2 in Nonhuman Primates. New England Journal of Medicine *383*, 1544–1555. 10.1056/NEJMOA2024671/SUPPL_FILE/NEJMOA2024671_DISCLOSURES.PDF.
29. Sanyal, G., Särnefält, A., and Kumar, A. (2021). Considerations for bioanalytical characterization and batch release of COVID-19 vaccines. Preprint at Nature Research, 10.1038/s41541-021-00317-4 10.1038/s41541-021-00317-4.

30. Rahman, M.M., Zhou, N., and Huang, J. (2021). An Overview on the Development of mRNA-Based Vaccines and Their Formulation Strategies for Improved Antigen Expression In Vivo. 10.3390/vaccines9030244.

31. Nourafkan, E., Gao, H., Hu, Z., and Wen, D. (2017). Formulation optimization of reverse microemulsions using design of experiments for nanoparticles synthesis. Chemical Engineering Research and Design *125*, 367–384. 10.1016/j.cherd.2017.07.023.
