# Supplementary material for: Quality by design for mRNA platform purification based on continuous oligo-dT chromatography
Source: Mol Ther Nucleic Acids. 2024 Sep 11;35(4):102333. doi: 10.1016/j.omtn.2024.102333 (PMC11458983; doi:10.1016/j.omtn.2024.102333)
Supplement: Document S2. Article plus supplemental information [file mmc2.pdf]

Molecular Therapy
Nucleic Acids
Original Article

Quality by design for mRNA platform purification based on continuous oligo-dT chromatography

Jixin Qu,[1] Adithya Nair,[1] George W. Muir,[1] Kate A. Loveday,[1] Zidi Yang,[1] Ehsan Nourafkan,[1] Emma N. Welbourne,[1] Mabrouka Maamra,[1] Mark J. Dickman,[1] and Zoltán Kis[1,2]

[1]School of Chemical, Materials and Biological Engineering, University of Sheffield, Sir Robert Hadfield Building, Mappin Street, Sheffield S1 3JD, UK; [2]Department of Chemical Engineering, Imperial College London, Roderic Hill Building, South Kensington Campus, London SW7 2AZ, UK

Oligo-deoxythymidine (oligo-dT) ligand-based affinity chromatography is a robust method for purifying mRNA drug substances within the manufacturing process of mRNA-based products, including vaccines and therapeutics. However, the conventional batch mode of operation for oligo-dT chromatography has certain drawbacks that reduce the productivity of this process. Here, we report a new continuous oligo-dT chromatography process for the purification of *in vitro* transcribed mRNA, which reduces losses, improves the efficiency of oligo-dT resin use, and intensifies the chromatography process. Furthermore, the quality by design (QbD) framework was used to establish a design space for the newly developed method. The optimization of process parameters (PPs), including salt type, salt concentration, load flow rate and mRNA load concentration both in batch and the continuous mode, achieved a greater than 90% yield (mRNA recovery) along with greater than 95% mRNA integrity and greater than 99% purity. The productivity of continuous chromatography was estimated to be 5.75-fold higher, and the operating cost was estimated 15% lower, when compared with batch chromatography. Moreover, the QbD framework was further used to map the relationship between critical quality attributes and key performance indicators as a function of critical process parameters and critical material attributes.

INTRODUCTION

mRNA technology is advancing the development of vaccines and therapeutics against a wide range of diseases, and the demand for scalable manufacturing processes that are efficient and cost effective is ever growing.[1] The cell-free production of mRNA vaccines and therapeutics has the advantages of simplicity, scalability and potential for affordability compared with conventional techniques.[2] Furthermore, it is considered a platform technology, as different mRNA-based vaccines and therapeutics against a wide range of diseases can be produced using the same production process, raw materials (except template DNA), standard operating procedures, and analytical methods.[3] This versatility highlights the potential of mRNA technology in responding rapidly to emerging health crises.[4] A faster, more scalable and economical manufacturing technique is required to address future pandemics and save lives.[5,6] One promising solution for boosting manufacturing productivity is to develop a continuous production process for mRNA vaccines and therapeutics.[7] As illustrated in Figure 1A, the continuous manufacturing process starts from the continuous *in vitro* transcription (IVT) reaction followed by the continuous downstream purification. Once purified, mRNA is sterile filtered, encapsulated within lipid nanoparticles (LNPs), purified by tangential flow filtration, and sterile filtered again before the fill-to-finish stage.[8]

Oligo-deoxythymidine (Oligo-dT) affinity chromatography is a robust process that purifies mRNA from a solution such as the IVT reaction mixture.[9] This technology leverages the selective binding affinity of short sequences (oligomers) of dT to the polyadenylated (poly[A]) tails present on mRNA molecules, facilitating efficient separation from the other nucleic acids.[10] As shown by the chromatography process schematic in Figure 1B, the mRNA is bound to the oligo-dT matrix when the IVT mixture is loaded onto the column under high salt concentration and off-target molecules are washed away with the flow-through, after which mRNA is eluted under low or no salt conditions. The oligo-dT affinity chromatography process has several advantages, including high recovery, compatibility with a wide range of mRNA sequences, scalability, and simplicity.[9] Salts such as sodium chloride (NaCl) play a crucial role in mRNA binding to the oligo-dT ligands. The cations from the salt suppress the electrostatic repulsions between the negatively charged phosphate backbone of mRNA and oligo-dT as it provides shielding between the negative charges of phosphates and facilitates hydrogen bonding.[11] The concentration is usually optimized to ensure the binding efficiency between the mRNA and oligo-dT matrix; a low salt concentration provides insufficient ionic strength to stabilize the hybridization,

Received 2 February 2024; accepted 5 September 2024;
https://doi.org/10.1016/j.omtn.2024.102333.

Correspondence: Zoltán Kis, School of Chemical, Materials and Biological Engineering, University of Sheffield, Sir Robert Hadfield Building, Mappin Street, Sheffield S1 3JD, UK.
E-mail: z.kis@sheffield.ac.uk

Published by Elsevier Inc. on behalf of The American Society of Gene and Cell Therapy.
This is an open access article under the CC BY license (http://creativecommons.org/licenses/by/4.0/).

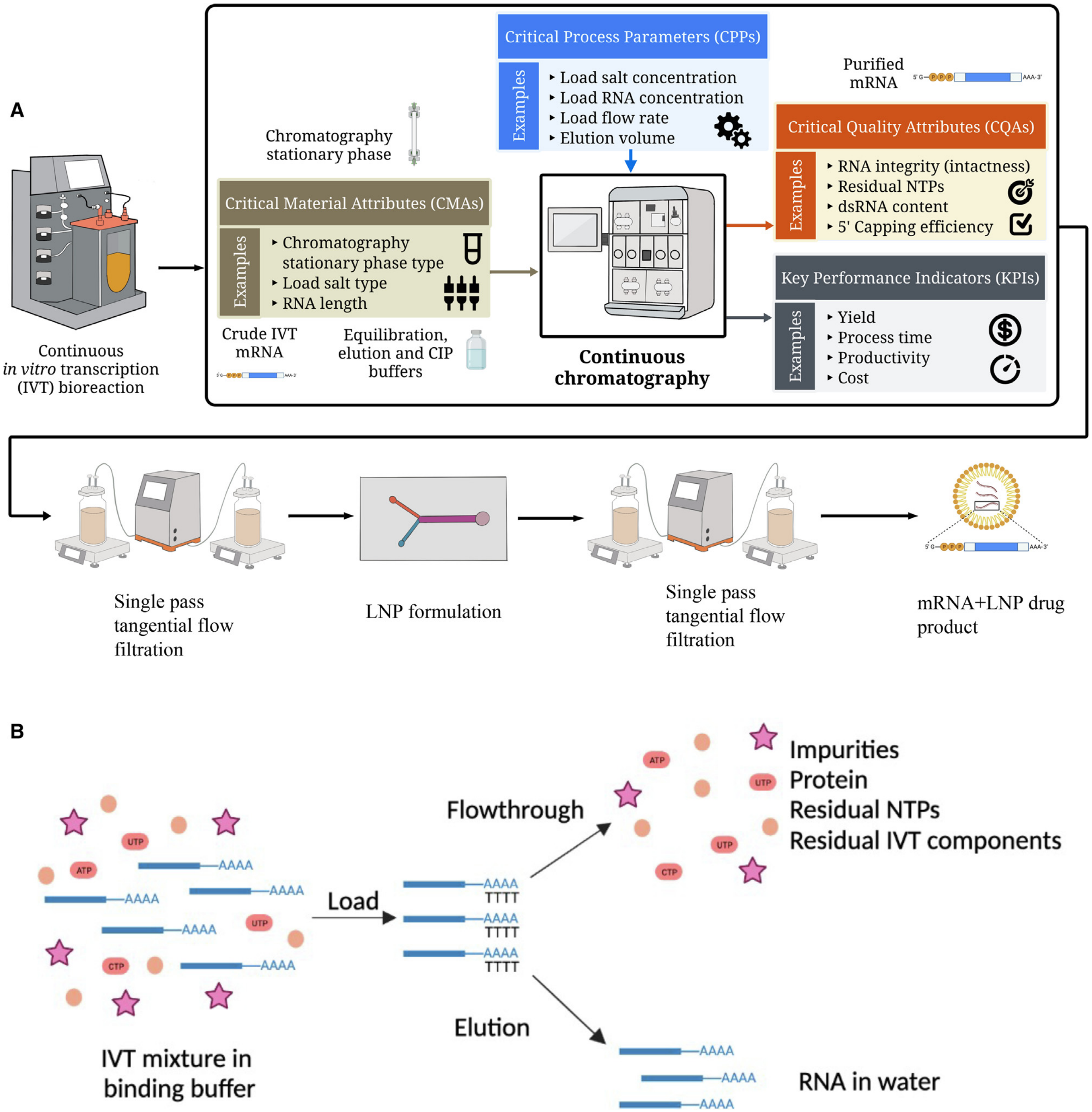

Figure 1. Overview of the proposed mRNA-based vaccines and therapeutics manufacturing process and the oligo-dT affinity purification method
(A) Illustration of the continuous mRNA-based vaccines and therapeutics manufacturing platform process with examples of CMAs, CPPs and CQAs for the continuous chromatography process. (B) Schematic of the oligo-dT affinity chromatography process.

resulting in poor binding. In contrast, a high salt concentration reduces the specificity of mRNA binding, allowing non-specific binding of other RNA molecules.[12] Moreover, high salt concentration can also precipitate mRNA.[9] It is reported in the literature that several potential chaotropic agents and salts such as guanidine hydrochloride (Gu-HCl), urea, and Tween 20 significantly promote the binding capacity of mRNA onto the oligo-dT column.[9] The addition of chaotropic agents disrupts the formation of the secondary structure of mRNA and facilitates the accessibility of the poly(A) tail to the oligo-dT ligand. Chaotropic agents affect the hydration shell of the mRNA molecules, leading to decreased water-mediated interactions between mRNA and other molecules, enhancing the binding capacity of

mRNA to the oligo-dT matrix.[9,13] However, the impact of using chaotropic agents in the purification processes on the safety and efficacy of mRNA vaccines and therapeutics (final product) is still under evaluation, as very limited literature has given conclusive results.[14]

Over the last few decades, continuous bioprocessing has shown many advantages in reducing the cost of manufacturing, increasing productivity, and allowing for significantly smaller scale equipment and facilities.[15] The focus of the continuous manufacturing process has primarily been directed at the development of upstream processes.[16–19] It is critical to extend the attention to the downstream purification processes, especially the continuous chromatography techniques, as they play a crucial role in the continuous manufacturing process to connect the upstream mRNA synthesis and the final formulation (e.g., LNP encapsulation). Continuous or semi-continuous chromatography as a downstream purification process has been developed and reported for several biological products, like, monoclonal antibodies[20–24] and other proteins.[16,25,26] Nevertheless, the literature has never reported any continuous chromatographic method for the downstream purification of mRNA-based vaccines and therapeutics.

To optimize and determine the design space of the chromatography process, the quality by design (QbD) framework has been applied. QbD is now considered one of the most promising approaches in pharmaceutical manufacturing development because quality is built into the product-process design and controlled during process operation based on robust scientific understanding.[27] Additionally, it maximizes the quality of products for their entire life cycle and allows potential changes in processes for further optimization even after regulatory approval, provided they are within the approved design space.[28] The potential of mRNA as a platform technology for therapeutics and vaccines is a recent development; as a result, major gaps remain in the validation of the multi-product design space to ensure the mRNA products' quality, safety, and efficacy.[29] Moreover, the combination of the mRNA platform technology and the QbD framework can lead to accelerated product development, regulatory approval, and the mass manufacturing of mRNA-based products in a disease-agnostic manner.[30] Importantly, the list of critical quality attributes (CQAs) for mRNA vaccines is independent of the disease target, although their acceptance ranges might change in a disease and product-specific manner. The disease-agnostic CQA list and associated analytical methods can further streamline the development of mRNA vaccines and therapeutics against a wide range of diseases. The QbD framework has been applied to IVT[31] and the LNP formulation processes.[32] Interestingly, the benefits of integrating the QbD framework with mRNA downstream purification unit operations have not yet been showcased in the literature.

In this study, a multi-column continuous oligo-dT affinity chromatography process was developed with the help of the QbD framework. The QbD framework is used to establish a design space for purifying a variety of mRNA sequences that can translate to different mRNA products. The QbD framework also guides the integration of the continuous platform chromatography unit operations into the development of a fully continuous manufacturing process (Figure 1A) for a wide range of mRNA vaccines and therapeutics.

RESULTS

QbD framework for the chromatography process

The application of QbD for a manufacturing process starts with the identification of product CQAs derived from the quality target product profile based on the patient's needs. CQAs are determined based on prior knowledge, preclinical and clinical data using a risk assessment scoring. After CQA identification, the next step is to link CQAs with critical process parameters (CPPs) of the manufacturing process to define the design space. This is supported by a combination of prior knowledge, product-process understanding, experimental product and process development data, expert knowledge and computational models. The list of CQAs was adapted from Daniel et al.[30] (2022), as shown in Tables S2–S4. In addition to CQAs, key performance indicators (KPIs) (e.g., manufacturing costs and productivity) are also quantified as a function of critical material attributes (CMAs) and CPPs. With this approach, a design space of CPPs and CMAs can be established in which we can obtain the optimal CQA and key performance indicator (KPI) values. The assessment criteria for the uncertainty and impact of CPPs and CMAs on CQAs and KPIs is shown in Table S5, where the impact score ranges from no impact (indicated by 0) to high impact (indicated by 3) and the uncertainty rating ranges from ^1 (indicated very low) and ^4 (indicated high). If a PP has an impact of no larger than 0 or 1 on any of the CQAs or KPIs, that PP is considered non-critical. However, if the PP has an impact of 2 or 3 on any of the CQAs or KPIs, that PP is considered critical, therefore a CPP,. The shape of the trend when plotting CQAs and KPIs as a function of PPs and CMAs is captured by + for a linear positive slope, by − for a linear negative slope, by ± for a non-linear peak-type behavior with an initial increase, reaching a maximum, followed by a decrease, and by ∓ for a non-linear valley-type behavior (inverse of the peak-type behavior) with an initial decrease, reaching a minimum followed by an increase.

The assessment of the impact of PPs and CMAs on CQAs and KPIs is shown in Tables 1 and 2 for the batch and continuous oligo-dT chromatography unit operations, respectively. This provides a comprehensive overview of how product CQAs, as well as manufacturing KPIs, can be controlled by adjusting PPs and selecting materials with adequate CMAs (when options exist). Further, it also facilitates the development and operation of manufacturing processes for the production of a wide range of mRNA vaccines and therapeutics at the required high quality and with the optimal manufacturing KPIs (e.g., high productivity and low cost). Despite the limited knowledge of the large-scale continuous manufacturing process, a number of in-process critical parameters can still be recognized due to the enhanced mechanistic understanding of the continuous chromatography techniques developed for other applications and the relevant digital twins.[5,23–26]

Table 1. Assessment of the criticality and impact of CPPs in combination with CMAs on the CQAs and KPIs of the batch oligo-dT chromatography unit operation for purifying a wide range of mRNA sequences

		PP or CMA										
		CPP	CMA		CPP	CPP	CPP	CPP	CPP	CPP	CMA	
CQA or KPI		Loading salt concentration	Loading salt type[a]	CPP	pH of load buffer	Load flow rate	Wash buffer volume	Elution volume	Elution flow rate	Temperature	mRNA length	References
KPI	mRNA yield [mg]	±3^1	±3^1	±2^1	±2^3	−3^1	±1^2	+1^4	−1^4	±1^4	−2^2	Mencin et al., 2023[9]; Cui et al., 2023[33]
KPI	Eluted mRNA amount [mg]	±3^1	±3^1	±2^1	±2^3	−2^1	−1^2	+1^4	−1^4	±1^4	−2^2	Mencin et al., 2023[9]; Cui et al., 2023[33]
CQA	mRNA sequence integrity	−1^3	±1^3	0^3	±2^4	−1^3	+1^4	+1^4	−1^4	±2^4	−1^3	Grinsted et al., 2022[34]; Gomis-Fons et al., 2020[35]; Kuribayashi et al., 1998[36]
CQA	mRNA structure integrity	±1^3	±1^3	0^3	±1^4	−1^3	+1^4	+1^4	−1^4	±2^4	−1^3	Grinsted et al., 2022[34]; Gomis-Fons et al., 2020[35]; Kuribayashi et al., 1998[36]
CQA	mRNA purity	±1^3	±1^1	−1^3	±2^4	−2^2	3^3	0^4	0^4	±1^4	0^3	Grinsted et al., 2022[34]; Gomis-Fons et al., 2020[35]; Kuribayashi et al., 1998[36]
CQA	5′ Capped mRNA percentage	−1^4	±1^3	0^4	±2^4	−1^4	2^4	0^4	−1^4	±1^4	−1^4	Mencin et al., 2023[9]; Korenč et al., 2021[12]
CQA	3′ Poly-A tail length and level	−1^4	±1^3	±1^4	±1^4	−1^4	2^4	0^4	−1^4	±1^4	−1^4	Mencin et al., 2023[9]; Korenč et al., 2021[12]
CQA	IVT residuals	∓2^4	∓2^3	2^3	∓2^4	1^2	−2^4	0^4	0^4	±1^4	0^2	Tiwari et al., 2023[8]; Nag et al., 2022[20]; Feng et al., 2022[37]; Lukas Vetter et al., 2022[38]
CQA	dsRNA concentration	−1^4	±1^3	+1^3	±1^4	−1^2	−2^4	−1^4	−1^4	±2^4	±1^2	Baiersdörfer et al., 2019[39]; Eskelin et al., 2022[40]
CQA	Shorter RNA species	±1^4	±1^3	2^3	±2^4	−2^1	−2^4	0^4	0^4	±1^4	−1^3	Tiwari et al., 2023[8]; Nag et al., 2022[20]; Feng et al., 2022[37]; Lukas Vetter et al., 2022[38]
CQA	RNA precipitation	3^1	±3^2	3^3	−3^1	0^4	−1^2	0^4	0^4	−1^4	0^2	Eon-Duval et al., 2003[41]
CQA	Immunogenicity	±3^3	±2^4	−2^4	±2^4	+1^3	2^4	+1^4	−1^4	±1^4	−1^4	Feng et al., 2022[38]; Eon-Duval et al., 2003[41]; Cheng et al., 2021[42]
CQA	Potency	±2^3	±2^3	−1^1	±2^4	+1^1	2^4	+1^4	−1^4	±1^4	−1^4	Tiwari et al., 2023[8]; Nag et al., 2022[20]; Feng et al., 2022[37]; Lukas Vetter et al., 2022[38]
CQA	Appearance	±1^1	±2^3	−2^1	0^4	0^1	+1^4	+1^4	−1^4	−2^4	0^2	Tiwari et al., 2023[8]; Nag et al., 2022[20]; Feng et al., 2022[37]; Lukas Vetter et al., 2022[38]
KPI	Process time [min]	0^1	0^3	−2^1	0^1	−3^1	3^1	3^2	−3^2	0^2	0^2	Mahajan et al., 2012[24]; Grinsted et al., 2022[34]; Girard et al., 2015[43]; Yao et al., 2006[44]

The definition of KPIs is included in Table S6.
[a]This is a categorical variable for which a peak trend was assumed when comparing the various salt types and chaotropic agents.

Table 2. Assessment of the criticality and impact of CPPs in combination with CMAs on the CQAs and KPIs of the continuous oligo-dT chromatography unit operation for purifying a wide range of mRNA sequences

		PP or CMA										
		CPP	CMA	CPP	CPP	CPP	CPP	CPP	CPP	CPP	CMA	
CQA or KPI		Loading salt concentration	Loading salt type[a]	mRNA load concentration	pH of load buffer	Load flow rate	Wash buffer volume	Elution volume	Elution flow rate	Temperature	mRNA length	References
KPI	mRNA yield [mg]	±3^1	±3^1	±2^1	±2^3	−3^1	±1^2	+1^4	−1^4	±1^4	−2^2	Mencin et al., 2023[9]; Cui et al., 2023[33]
KPI	Eluted mRNA amount [mg]	±3^1	±3^1	±2^1	±2^3	−2^1	−1^2	+1^4	−1^4	±1^4	−2^2	Mencin et al., 2023[9]; Cui et al., 2023[33]
CQA	mRNA sequence integrity	−1^3	±1^3	0^3	±2^4	−1^3	+1^4	+1^4	−1^4	±2^4	−1^3	Grinsted et al., 2022[34]; Gomis-Fons et al., 2020[35]; Kuribayashi et al., 1998[36]
CQA	mRNA structure integrity	±1^3	±1^3	0^3	±1^4	−1^3	+1^4	+1^4	−1^4	±2^4	−1^3	Grinsted et al., 2022[34]; Gomis-Fons et al., 2020[35]; Kuribayashi et al., 1998[36]
CQA	mRNA purity	±1^3	±1^1	−1^3	±2^4	−2^2	3^3	0^4	0^4	±1^4	0^3	Grinsted et al., 2022[34]; Gomis-Fons et al., 2020[35]; Kuribayashi et al., 1998[36]
CQA	5′ Capped mRNA percentage	−1^4	±1^3	0^4	±2^4	−1^4	2^4	0^4	−1^4	±1^4	−1^4	Mencin et al., 2023[9]; Korenč et al., 2021[12]
CQA	3′ Poly-A tail length and level	−1^4	±1^3	±1^4	±1^4	−1^4	2^4	0^4	−1^4	±1^4	−1^4	Mencin et al., 2023[9]; Korenč et al., 2021[12]
CQA	IVT residuals	∓2^4	∓2^3	2^3	∓2^4	1^2	−2^4	0^4	0^4	±1^4	0^2	Tiwari et al., 2023[8]; Nag et al., 2022[20]; Feng et al., 2022[37]; Lukas Vetter et al., 2022[38]
CQA	dsRNA concentration	−1^4	±1^3	+1^3	±1^4	−1^2	−2^4	−1^4	−1^4	±2^4	±1^2	Baiersdörfer et al., 2019[39]; Eskelin et al., 2022[40]
CQA	Shorter RNA species	±1^4	±1^3	2^3	±2^4	−2^1	−2^4	0^4	0^4	±1^4	−1^3	Tiwari et al., 2023[8]; Nag et al., 2022[20]; Feng et al., 2022[37]; Lukas Vetter et al., 2022[38]
CQA	RNA precipitation	3^1	±3^2	3^3	−3^1	0^4	−1^2	0^4	0^4	−1^4	0^2	Eon-Duval et al., 2003[41]
CQA	Immunogenicity	±3^3	±2^4	−2^4	±2^4	+1^3	2^4	+1^4	−1^4	±1^4	−1^4	Feng et al., 2022[38]; Eon-Duval et al., 2003[41]; Cheng et al., 2021[42]
CQA	Potency	±2^3	±2^3	−1^1	±2^4	+1^1	2^4	+1^4	−1^4	±1^4	−1^4	Tiwari et al., 2023[8]; Nag et al., 2022[20]; Feng et al., 2022[37]; Lukas Vetter et al., 2022[38]
CQA	Appearance	±1^1	±2^3	−2^1	0^4	0^1	+1^4	+1^4	−1^4	−2^4	0^2	Tiwari et al., 2023[8]; Nag et al., 2022[20]; Feng et al., 2022[37]; Lukas Vetter et al., 2022[38]
KPI	Process time [min]	0^1	0^3	−2^1	0^1	−3^1	3^1	3^2	−3^2	0^4	0^2	Mahajan et al., 2012[24]; Grinsted et al., 2022[34]; Girard et al., 2015[43]; Yao et al., 2006[24,34,43,44]
KPI	Column switch interval [min]	2^2	±2^3	−2^3	0^3	+3^1	+1^2	2^2	−2^2	0^4	0^2	Tiwari et al., 2023[20]; Mendes et al., 2022[26]; Steinebach et al., 2016[20,26,45]
KPI	Phase time for continuity [min]	2^2	±2^3	−2^3	0^3	−3^1	+1^2	2^2	−2^2	0^4	0^2	Tiwari et al., 2023[20]; Mendes et al., 2022[26]; Steinebach et al., 2016[45]
KPI	Productivity [mg/min/mL]	±1^2	±2^3	±3^3	0^3	±2^1	−2^2	±1^2	±1^2	0^4	−1^2	Mencin et al., 2023[9]; Ng et al., 2014[46]; Tugcu et al., 2008[47]

The definition of KPIs is included in Table S6.
[a]This is a categorical variable for which a peak trend was assumed when comparing the various salt types and chaotropic agents.

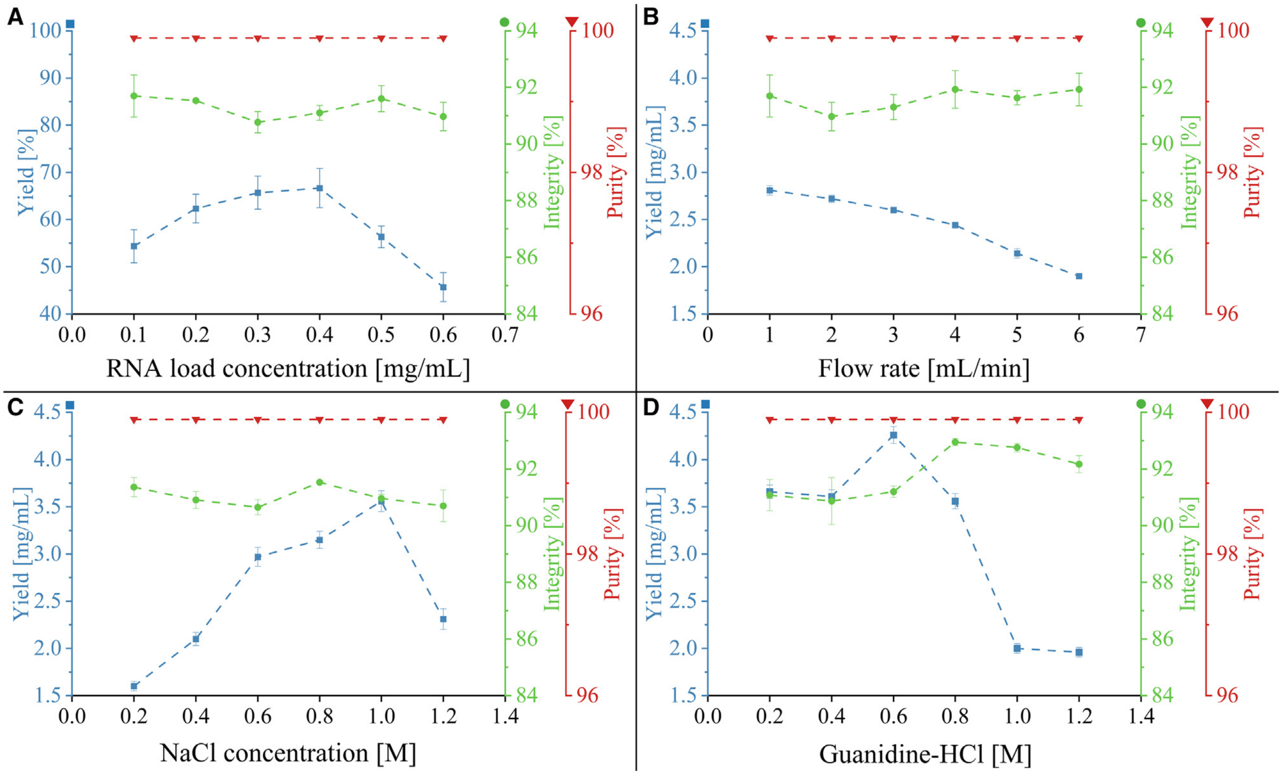

Figure 2. Oligo-dT chromatography results from one-factor-at-a-time ÄKTA PCC batch runs for assessing the impact of loading salt type CPP, loading salt concentration CPP, eGFP mRNA load concentration CPP and the flow rate CPP on the mRNA yield KPI (measured by AEX HPLC), mRNA integrity CQA (measured by CGE) and mRNA purity CQA (measured by AEX HPLC)

The mean CQA and KPI values are shown by dots, and error bars represent standard deviation for three replicates ($n = 3$). (A) Varied mRNA load concentration (mg/mL), under constant load flow rate of 1 mL/min, constant 0.6 M NaCl concentration in loading buffer, and a constant total loading volume of 10 mL. (B) Varied flow rate (mL/min), under constant mRNA load concentration of 0.4 mg/mL, constant 0.6 M NaCl concentration in loading buffer, and a constant total loading volume of 10 mL. (C) Varied NaCl salt concentration (M), under constant mRNA load concentration of 0.6 mg/mL in constant 10 mL load volume and constant flow rate of 1 mL/min. (D) Varied Gu-HCL salt concentration, under constant mRNA load concentration of 0.6 mg/mL in constant 10 mL load volume and constant flow rate of 1 mL/min.

One-factor-at-a-time experimental screening of mRNA yield, integrity, and purity as a function of mRNA load concentration, load flow rate, loading salt type, and loading salt concentration in batch mode

We investigated a subset of changes in CQAs and KPIs as a function of CPPs and CMAs. The CPPs, such as mRNA load concentration, load flow rate, loading salt concentration, and the CMA loading salt type, were tested against yield KPI and the CQAs mRNA integrity and mRNA purity. Previous studies have demonstrated these CPPs and CMAs as the most significant and influential factors for the quality of purification by chromatography.[9,34,48]

Experiments were performed in triplicate for each condition to determine the relationship of CQAs-CPPs. As shown in Figure 2A, the eGFP mRNA load concentration was varied from 0.1 to 0.6 mg/mL, based on 1–6 mg of mRNA in a 10-mL total load volume, at a load flow rate of 2 mL/min and 600 mM NaCl load concentration. Under these conditions, the yield (%) increased when the mRNA load concentration ranged from 0.1 to 0.4 mg/mL and decreased when it was more than 0.4 mg/mL. The highest yield at 0.4 mg/mL load concentration, corresponding with 4 mg of mRNA loaded per milliliter of column resin was expected because of the maximum binding capacity for the specific oligo-dT column (4 mg) for the specified PPs (based on information from the column manufacturer). At higher mRNA loading amounts (e.g., 5 or 6 mg), the excess mRNA above the column binding capacity is unable to bind to the oligo-dT stationary phase. On the other hand, mRNA integrity showed minimal variation in the 90%–92% range, with greater than 99% purity for all analytes, as no NTPs were detected by anion exchange high-performance liquid chromatography (AEX HPLC). Moreover, the impact of the load flow rate was investigated by varying it from 1 to 6 mL/min with 600 mM NaCl as the loading salt. As results show in Figure 2B, the yield was consistent when the flow rate ranged from 1 to 3 mL/min, but at flow rates of greater than 3 mL/min, a negative slope was observed, possibly due to the reduced residence time for the binding to happen between the oligo-dT matrix and poly-A tailed mRNA at these higher flow rates.[34] Similar to the previous runs, the integrity of all analytes was approximately 90%–91%, with a purity of greater than 99%.

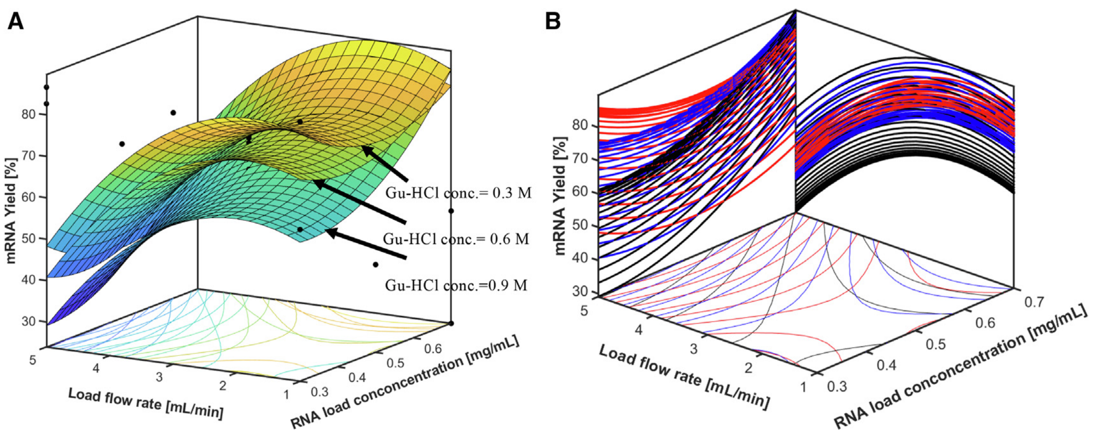

Figure 3. Results from multi-factorial optimisation of mRNA yield

(A) Three-dimensional surface plot for the Oligo-dT chromatography multi-factorial results from ÄKTA PCC batch runs for assessing the impact of Gu-HCl load salt concentration CPP, mRNA load concentration CPP, and load flow rate CPP on mRNA yield KPI (measured by AEX HPLC). (B) Two-dimensional projection plots of (A) onto the side, rear, and bottom planes. Red lines, Gu-HCl concentration at 0.3 M; blue lines, Gu-HCl concentration at 0.6 M; black lines, Gu-HCl concentration at 0.9 M.

The literature indicated that loading salt concentration and loading salt type can have significant impacts on the yield, and by using a chaotropic agent such as Gu-HCl as the replacement for NaCl, the binding capacity and, as a result, the final yield can be significantly improved.[9,13,25] Thus, two types of salts in the load buffer, NaCl and Gu-HCl concentrations, varied from 0.2 to 1.2 M, while other components were kept constant in the load buffer. As known from the previous tests, the maximum binding capacity of the 1-mL monolith oligo-dT column is approximately 4 mg. All runs were performed by overloading the column with 6 mg crude mRNA at the concentration of 0.6 mg/mL to determine the maximum yield with varied salt concentration and different salt types. As shown in Figures 2C and 2D, the yield increased until 0.6 M for Gu-HCl and 1 M for NaCl. IVT crude products loaded with Gu-HCl have generally given higher yields compared with NaCl-based buffers. The enhanced mRNA binding observed for Gu-HCl-based buffers is likely due to the chaotropic agent disrupting the hydration shell around charges and polar groups, reducing the hydrophobic interactions, thus exposing poly(A) tail on the mRNA to the oligo-dT matrix.[49] Furthermore, with Gu-HCl, the binding capacity of the monolith column can be pushed to above 4 mg/mL. Presumably, a higher yield should be achievable in continuous chromatography, where the first column in the loading zone can be loaded with greater amounts than in batch mode without losing mRNA, as the breakthrough can be captured by the second column in the loading zone. This enables the reduction of losses while also utilizing more of the column binding capacity. The 600 mM Gu-HCl has shown the highest yield with a mean of 4.17 mg and a SD of 0.09 mg; thus, this concentration was used as the loading condition for the continuous chromatography runs. All eluted samples have given an mRNA integrity of more than 90% and a purity of greater than 99%, without detectable impurities using the AEX HPLC method. As 20 min were costed, 1 mL chromatographic resin was applied, and 4.17 mg of mRNA was purified; the productivity was calculated as 0.21 mg/min/mL.

In summary, the mRNA load concentration CPP has a relatively high impact on the yield KPI, but a limited impact on the mRNA integrity and the mRNA purity CQAs. In contrast, increasing the flow rate CPP will negatively affect the yield KPI as it decreases the binding interaction time between the oligo-dT matrix and the mRNA. However, this CPP has minimal effects on the mRNA integrity and purity CQAs. Furthermore, salt concentration CPP and salt type CMA have significant impacts on the yield KPI but minimal effects on mRNA integrity and purity CQAs.

Multi-factorial optimization of mRNA yield, integrity, and purity as a function of the combination of load salt concentration, mRNA load concentration, and load flow rate in batch mode

As shown in Figure 2, using Gu-HCl as the loading salt showed a generally greater binding capacity and resulted in a higher yield with preferable mRNA integrity and purity. Thus, it is worth investigating the impacts of the combination of the following three CPPs, load salt concentration of Gu-HCl, mRNA load concentration, and load flow rate on the yield KPI and mRNA integrity and purity CQAs. A Design of Experiments (DoE) was performed to vary three factors at three levels, which included Gu-HCl concentrations of 0.3, 0.6, and 0.9 M, eGFP mRNA load concentrations of 0.3, 0.5, and 0.7 mg/mL in a 10-mL load, and load flow rates of 1, 3, and 5 mL/min. In total, 16 runs were performed in triplicates using the batch chromatography method, and 20 mL elution was collected for each test. Elution fractions were analyzed by AEX HPLC for mRNA quantification and purity, and capillary gel electrophoresis (CGE) for mRNA integrity.

The AEX HPLC and CGE results show that all of the eluted mRNA analytes give greater than 99% mRNA purity and greater than 90% mRNA integrity. The three-dimensional surface plot in Figure 3 illustrates the result for yield. As expected, the increasing load flow rate leads to a lower yield under all investigated Gu-HCl concentrations

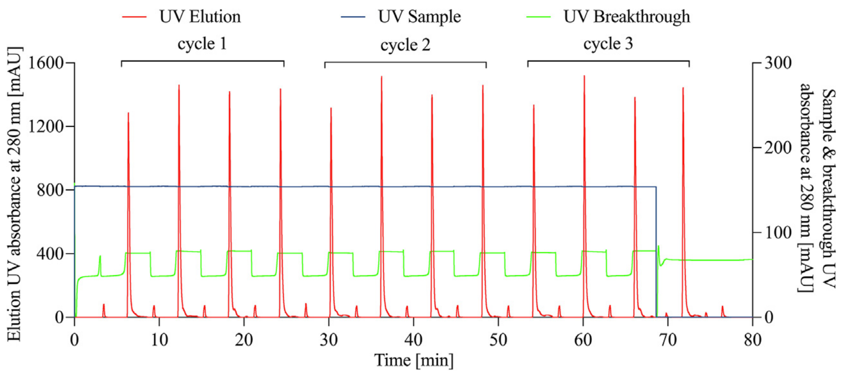

Figure 4. Chromatogram of the continuous oligo-dT results performed at load salt concentration of 600 mM Gu-HCl, mRNA load concentration of 0.23 mg/mL and load flow rate of 4 mL/min

In total, 3 cycles of 12 load-elutes were performed using 1 mL monolith oligo-dT columns, with a total run time of 70 min excluding the start-up equilibration and shut-down phases. In this run, 41.44 mg of mRNA was purified.

and mRNA load concentrations due to the reduced contact time between mRNA and the oligo-dT matrix. While load volume remained unchanged, increasing the mRNA load concentration with other two input factors constant generally lead to higher yield.[48] Moreover, with the load Gu-HCl concentration at the low level (0.3 M), the yield can reach more than 80% of the maximum value when the load flow rate is at a low or medium level (1 or 3 mL/min) and a low level of mRNA load concentration. However, the yield decreases slightly when the mRNA load concentration increases, because a higher flow rate and a higher mRNA load concentration decrease the contact time between mRNA and the oligo-dT matrix. With the conditions of medium or high level of Gu-HCl (0.6 or 0.9 M), the yield started comparably low when the corresponding mRNA load concentration was at a low level (0.3 mg/mL) and it improved significantly with the increase in mRNA load concentration (0.5 and 0.7 mg/mL) at a low or medium load flow rate (1 or 3 mL/min). A high salt concentration leads to a decrease in electrostatic repulsion between the mRNA and oligo-dT matrix, allowing more mRNA to be loaded to the oligo-dT column.[12] Interestingly, for medium level of Gu-HCl concentration (0.6 M) with mRNA load concentration above medium level (0.5 mg/mL) along with all-load flow rate conditions, the yield surpasses that of other runs with medium or high levels of Gu-HCl concentrations (0.6 and 0.9 M). The results indicate that the medium level of salt concentration is optimal, as a too-low salt concentration does not provide sufficient charge suppression between the negatively charged phosphate backbones of mRNA and oligo-dT matrix, and excessive salt can reduce the affinity of the oligo-dT to the mRNA due to the competition between the salt ions and the oligo-dT for interactions with poly(A) tails of mRNA.[13] At high salt concentrations, mRNA precipitation can also occur, reducing the amount of mRNA available to bind to the oligo-dT ligands. ANOVA was performed to determine the most significant input factor among three inputs. As shown in Equation S1 and Tables S7 and S8, the most significant input factor is mRNA load concentration, which has the highest F value and lowest *p* value.

These results indicate that mRNA load concentration CPP has a significant impact on the yield KPI for all tested salt types. The salt concentration CPP also has a significant impact on the yield KPI. Furthermore, load flow rate CPP has a medium-level impact on yield KPI, and its combinations with other CPPs, such as mRNA load concentration, lead to varied impacts on the yield KPI. Nevertheless, it is important to note that load salt concentration CPP, load flow rate CPP, and mRNA load concentration CPP only have minimal impacts on the mRNA purity and integrity CQAs.

Four-column continuous oligo-dT chromatography

Based on the batch optimization performed, the continuous oligo-dT affinity chromatography method was designed by adopting the optimal loading buffer of 600 mM Gu-HCl to demonstrate the possibility of using periodic counter-current chromatography (PCC) for continuous downstream purification of *in vitro* transcribed mRNA. eGFP mRNA (11 mg/mL) after an IVT reaction was diluted to 0.23 mg/mL with the optimized binding buffer and used in continuous chromatography; 20 mL was set to be loaded for each column with the flow rate of 4 mL/min. The flow rate was set at a higher value to reduce the running time for the initial tests, and this was to be optimized in the subsequent experiments. In total, 12 load-elute loops were performed, which utilized approximately 190 mL of the diluted load sample, as the Breakthrough UV absorbance at 280 nm guided the column switch.

As shown in the chromatogram in Figure 4, each cycle is composed of a four-column load-elute operation. The UV sample curve in blue shows a continued, non-stop loading for the entire process, benefiting the column switch mechanism described in the section above. The column switch interval is regulated by the UV breakthrough curve indicated in green, and the positions of the column switches move the load to the following columns when the curve reaches the breakthrough point. The chromatogram also showed a periodic and consistent elution, which was highlighted in red, where eluted eGFP mRNA was collected. The slight deviation of the peaks of UV elution in different loops was caused by the slight differences in the quality of the four columns, although the comparison should be made based on peak area rather than peak height. The loss of mRNA during the continuous chromatography run has been minimized to 6.5% (2.83 mg loss compared with the total load of 44.27 mg). This is due to (1) having two columns in the loading zone that allows capturing the breakthrough from the first column onto the second column, (2) the post-load wash that contains mRNA product flows to the third column, which at that stage has no mRNA bound and is ready to bind mRNA in the wash, and (3) the UV breakthrough signal after the first column in the loading zone is used to guide the

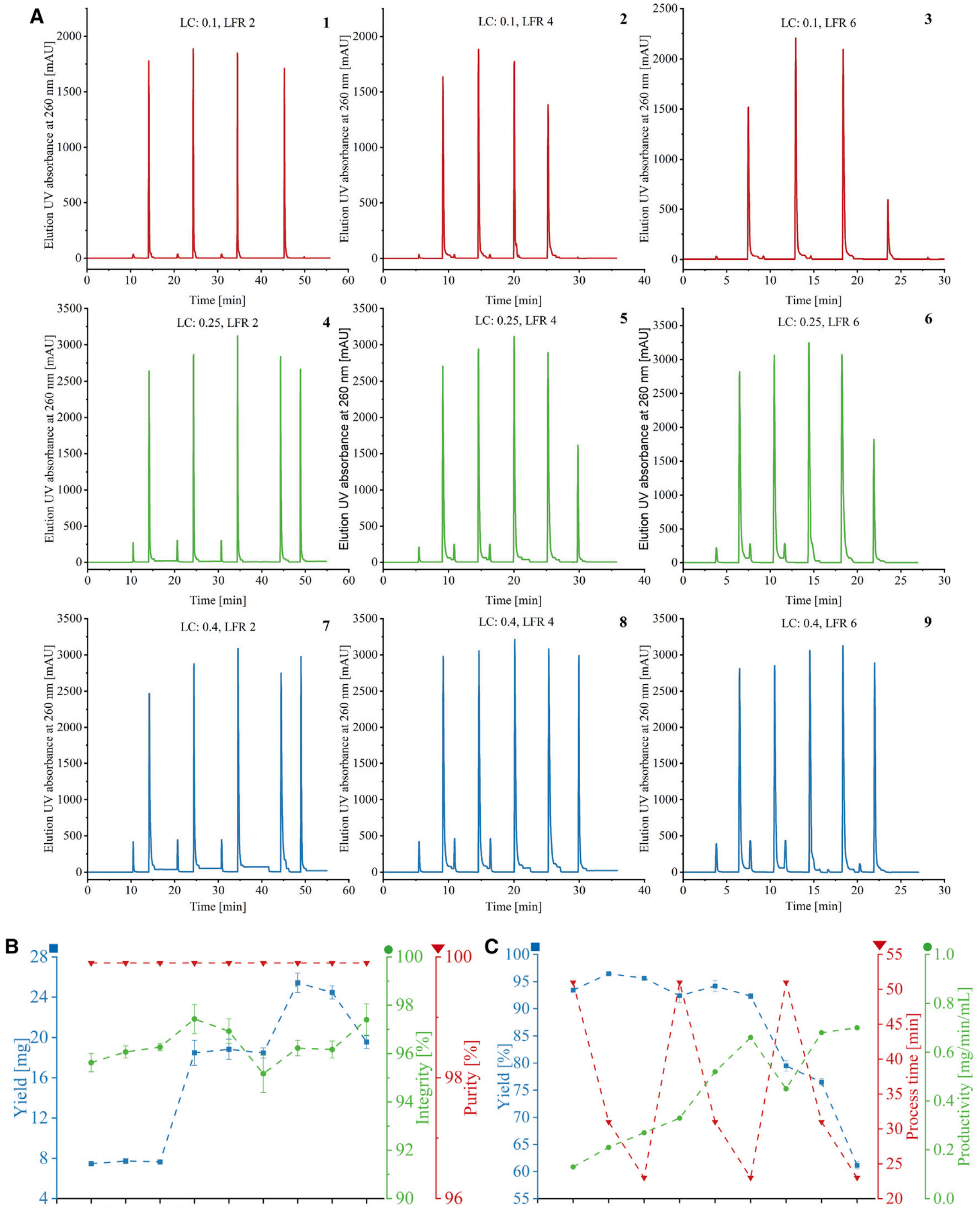

No. of Test	1	2	3	4	5	6	7	8	9
Load conc. [mg/mL]	0.1	0.1	0.1	0.25	0.25	0.25	0.4	0.4	0.4
Load flow rate [mL/min]	2	4	6	2	4	6	2	4	6

No. of Test	1	2	3	4	5	6	7	8	9
Load conc. [mg/mL]	0.1	0.1	0.1	0.25	0.25	0.25	0.4	0.4	0.4
Load flow rate [mL/min]	2	4	6	2	4	6	2	4	6
mRNA purified [mg]	7.47	7.72	7.65	18.48	18.83	18.47	25.43	24.47	19.57

(legend on next page)

column switch, ensuring optimal column loading amounts. Eluted samples of three cycles have been collected, corresponding to 12 load-elute loops. The results have shown a considerably high yield (93.62%) of 41.44 mg purified out of 44.27 mg load measured by AEX HPLC, greater than 95% mRNA integrity measured by CGE, and greater than 99% purity for all elution fractions (without detecting any NTP impurities), as measured by AEX HPLC. The flow-through was also collected, and it did not show the presence of mRNA. As the entire continuous chromatography process took 70 min, excluding the initial equilibration and shut-down phases, with only 1 mL of the chromatographic resin applied at a time, 41.44 mg of mRNA was purified, the productivity was calculated as 0.92 mg/min/mL, which is significantly higher than that the productivity (0.30 mg/min/mL) of the batch chromatography. Continuous chromatography runs were also performed to purify severe acute respiratory syndrome coronavirus 2 (SARS-CoV-2) Spike protein mRNA from unpurified crude IVT, see Figure S4, with similar CPPs, CQAs, and KPIs to eGFP mRNA continuous chromatography purification from unpurified crude IVT.

Four-column continuous oligo-dT chromatography for determining the multi-factorial relationship of load flow rate and mRNA load concentration with the process time, mRNA yield, mRNA integrity, mRNA purity, and productivity

Another DoE varying the eGFP mRNA load concentration at 0.1, 0.25, and 0.4 mg/mL in 80 mL load volume (20 mL for each column load) corresponding with load flow rates of 2, 4, and 6 mL/min in continuous chromatography was performed for testing the effects of flow rate and mRNA load concentration CPPs on process time, mRNA yield and productivity KPIs, along with mRNA integrity and purity CQAs. In total nine continuous chromatography runs have been completed in triplicates (each run comprised of four loops) with 600 mM Gu-HCl as the loading salt. As shown in the chromatogram of Figure 5A, for the first three tests with the load concentration of 0.1 mg/mL, only four elution peaks were observed. However, for tests three through nine, five elution peaks were observed even though only four loops had been programmed. This was due to higher loading concentrations that caused mRNA breakthrough into the second column in the loading zone, and the extra peak was the elution from the column, which captured the breakthrough.

As shown in Figure 5B, all eluted analytes have shown more than 95% mRNA integrity and greater than 99% purity as no NTPs have been detected, which indicates that load flow rate and mRNA load concentration CPPs have very little impact on mRNA integrity CQA and mRNA purity CQA. Nevertheless, as shown in the results of tests seven to nine, the mRNA yield decreased significantly when the load flow rate increased from 2 to 6 mL/min at high mRNA load concentrations of 0.4 mg/mL. This can be explained by (1) a reduced contact time between the mRNA and oligo-dT ligands at high flow rates and (2) column overloading with 8 mg mRNA per milliliter of column volume at high mRNA concentrations. The percentage yield, process time, and productivity are shown in Figure 5C; it is worth noting that the first six tests have shown yields of approximately 95%, with 0.1 and 0.25 mg/mL mRNA load concentrations; however, the percentage yield was greatly decreased when the mRNA load concentration was increased to 0.4 mg/mL. The process time of the continuous chromatography was only affected by the load flow rate, and it excluded the start-up equilibration and shut-down phases. The productivity was impacted by the process time and the amount of resin applied, only 1 mL of the chromatography resin was fully utilized during the continuous chromatography process. Notably, higher mRNA load concentration and higher flow rate led to generally higher productivity; however, the percentage yield was decreased significantly when the mRNA load concentration was at the high level (0.4 mg/mL) due to column overloading. ANOVA was performed as shown in Equation S1 and Tables S9 and S10, mRNA load concentration has a greater impact on yield compared with load flow rate. The productivity KPI, along with the other CQAs and KPIs, will guide further development and scale-up.

DISCUSSION

A multi-column continuous oligo-dT chromatography process is crucial for mitigating the shortcomings related to productivity observed in a single-column oligo-dT chromatography process operated in batch mode. Moreover, it will serve as the vital link between the continuous synthesis and formulation processes in an integrated/end-to-end closed manufacturing system. A continuous mRNA manufacturing system complements the inherent platform's ability to produce mRNA-based products. Such a manufacturing platform with a fully defined design space based on the QbD framework can also accelerate the regulatory approval process. In this study, a continuous oligo-dT chromatography system was developed for the downstream purification of *in vitro*-transcribed mRNA and showed more than 90% yield, 95% mRNA integrity, and greater than 99% purity, which matched the same CQAs of mRNA purified using oligo-dT chromatography.[9,50] The improvements seen in the cost and productivity KPIs are because for each bind-elute cycle, a maximum of approximately 5 mg of mRNA can be purified per milliliter of oligo-dT resin using continuous chromatography. In comparison, only approximately 4 mg of mRNA per mL of oligo-dT resin can be purified in batch. Moreover, the productivity has also improved by a factor of 3 when transitioning from batch to continuous chromatography. The highest productivity obtained in batch mode after

Figure 5. Oligo-dT chromatography chromatogram from ÄKTA PCC continuous runs for assessing the impact of mRNA load concentration CPP and the load flow rate CPP on the mRNA yield KPI (measured by AEX HPLC), mRNA integrity CQA (measured by CGE) and mRNA purity CQA (measured by AEX HPLC)

The load volume was 80 mL (20 mL for each column load-elute) per test. The mean CQA and KPI values are shown by dots, and error bars represent standard deviation for three replicates ($n = 3$) of tests 1–9. (A) Chromatograms of the 9 oligo-dT continuous chromatography tests. LC, load concentration (mg/mL); LFR, load flow rate (mL/min). (B) Yield (mg), mRNA integrity (%), and mRNA purity (%) of the nine tests. (C) Yield (%), process time (min), and productivity (mg/min/mL) of the purification runs.

optimization was 0.30 mg/min/mL, while the highest productivity obtained in continuous mode after optimization was 0.92 mg/min/mL with a more than 90% yield. The increase in productivity of the continuous chromatography is significantly higher than that of the batch run if the purification is performed continuously for a longer time. If we compare the productivity for a 24-h period, the continuous chromatography productivity at 0.92 mg/min/mL is 5.75-fold higher than the batch at 0.16 mg/min/mL (see supplemental material section 1.1 for details). This is due to the start-up equilibration and shutdown CIP requirement for each run in batch mode and the preparation time of 5 min between batch runs. By assessing the operating costs of the chromatography unit operation, it is estimated that a 15% operating cost reduction is achievable when transitioning from batch to continuous chromatography (see supplemental material section 1.2 for details). Additional savings of fixed costs can be realized in continuous chromatography mode due to (1) increased productivity therefore reduced facility footprint and reduced facility cost requirements, as well as (2) reduced labor requirements due to automation available in continuous mode. The cell transfection work was performed to demonstrate the functionality of the purified mRNA, the details are given in section 1.3 of the Supplemental Material and Figure S1. The results have shown that the mRNA purified by the batch and continuous chromatography expressed proteins in cultured cells.

Moreover, a QbD framework was established to assess the oligo-dT chromatography unit operation both in batch and in continuous modes for investigating the impact of CPPs and CMAs on CQAs and KPIs. The productivity KPI is affected by the process time, yield and the resin applied, higher load flow rate and mRNA load concentration generally lead to higher productivity, however, increasing flow rate and mRNA load concentration may lead to a reduced percentage yield. Thus, it is imperative that the productivity KPI and the yield KPI are considered concurrently to ensure a comprehensive assessment. The further characterization of CMAs, CPPs, CQAs, and KPIs will follow an iterative cycle based on the QbD framework.

The oligo-dT chromatography method is versatile and it was utilized to purify multiple mRNA sequences in batch mode.[9,11,51,52] In our work, oligo-dT chromatography translated well from batch to continuous and was used to purify both eGFP mRNA (c.f. Figures 2, 3, 4, and 5) and SARS-CoV-2 Spike protein mRNA (c.f. Figure S3 in the Supplemental Material). Similar continuous chromatography processes were adapted from batch from batch to continuous for the purification of proteins and other biopharmaceuticals.[43,53–57] Therefore, we consider that continuous multi-column oligo-dT is suitable for purification of a wide range of mRNA molecules that contain a polyA tail directly from unpurified crude IVT. As a consequence, continuous oligo-dT chromatography can be considered as a platform process for the multi-sequence, multi-product mRNA vaccine and therapeutics manufacturing downstream purification. Moreover, variation of the IVT composition around the yield-optimal conditions did not impact the performance of the optimized batch or continuous oligo-dT chromatography. The true benefits of the continuous chromatography process will be realized when the entire manufacturing process is operated continuously, rather than only a single continuous unit operation in isolation. This is because in a continuous manufacturing process, all unit operations are used simultaneously in sync with each other, contrary to conventional batch manufacturing when most unit operations are idle or underutilized, further compounded by the holdups of batch-release testing. The advantages of the continuous manufacturing process also include the flexibility in production throughput with less scaling up/down requirements, greater productivity, steady state optimal operation, lower footprint for equipment and facility, and suitability for closed system integration.[58]

However, there are challenges of continuous multi-column chromatography relative to batch chromatography, including (1) more complex equipment with intricate flow paths, (2) higher equipment purchase costs, and (3) the need for highly trained operators to run the process. Nevertheless, fewer operators are required per unit amount of purified mRNA for continuous chromatography compared with batch chromatography due to the greater productivity and increased automation of continuous chromatography.

In future work, additional quantification of process-related and product-related impurities will be beneficial. The purity of mRNA in this study was assessed using a new and rapid HPLC method developed in-house[59] based on quantifying the percentage of mRNA relative to NTPs and template DNA using UV detection. Process-related impurities worth assessing and quantifying in future work include (1) residual enzymes and proteins (e.g., T7 RNA polymerase, inorganic pyrophosphatase, RNAse inhibitors, *E. coli* host cell proteins introduced with protein and template DNA material produced in *E. coli*), (2) endotoxins originating from protein and template DNA material produced in *E. coli*, and (3) low levels of residual template DNA and host cell DNA carried over from *E. coli*.[60] The use of enzymatically synthesized template DNA might reduce the amount of process-related impurities or change the impurity profile. A more challenging purification problem is the removal of product-related impurities, which include immunogenic double-stranded RNA, abortive transcripts, truncated RNA, and RNA-DNA hybrids.[60] The removal of dsRNA impurities is especially important for mRNA therapeutic applications.[39,61,62] For this, more sensitive analytical methods are needed with lower limits of detection and lower limits of quantification. In-line or on-line real-time process analytical technology for CQA and KPI monitoring and control would further improve the continuous chromatography purification of mRNA.

Further development will involve scaling up the chromatography process and testing the robustness of the newly developed process, which will be aided by the QbD framework and the set of CQAs and KPIs presented here. It is also worth mentioning that this study has not fully validated all CQAs or KPIs and CPPs or CMAs listed in the QbD framework and their relationships due to limitations of resources. However, this research has tested the most significant and direct factors that affect the quality of mRNA-based products. More tests, including lab and in-silico experiments can be performed to

further validate the QbD framework presented here. It is important to note that no mRNA vaccines have been developed until now under a fully validated QbD framework, but the increased technological and computational capabilities are now paving the way toward a cost-effective, transferable, fully continuous platform production process for mRNA vaccines and therapeutics.

MATERIALS AND METHODS

mRNA transcripts

The IVT reaction was performed using previously described components.[63,64] The eGFP mRNA transcript consisting of 995 nucleotides (nt) and SARS-CoV-2 Spike protein mRNA transcript consisting of 4284 nt were prepared using IVT with the linearized eGFP and SARS-CoV-2 Spike protein plasmid template DNA provided by Genscript Biotech Corporation. In addition to template DNA, IVT reactions utilized, T7 bacteriophage DNA-dependent RNA polymerase (Takara Bio Europe; Roche; New England Biolabs) and ribonucleotide triphosphates ATP, CTP, GTP, and UTP (Roche; New England Biolabs) ($\leq$40 mM total). The reaction was further supplemented with magnesium acetate (Sigma-Aldrich), HEPES buffer (pH 7) (Roche), dithiothreitol (Roche), NaCl (Sigma-Aldrich), and spermidine (Roche). Inorganic pyrophosphatase (Roche; New England Biolabs) was also added to the reaction to prevent magnesium pyrophosphate precipitation. RNase inhibitor (Roche; New England Biolabs) was added to maintain an RNase-free environment. The IVT reaction was incubated at 37°C for 2 h, after which it was quenched by adding 200 mM EDTA. After IVT, the mRNA was purified for an initial check by solid phase extraction using Monarch RNA Cleanup kit (New England Biolabs), followed by the measurement of mRNA concentration using the NanoDrop OneC spectrophotometer (Thermo Fisher Scientific) by measuring absorbance at 260 nm with auto-ranging pathlength in the 0.030–1.0 mm range.[65]

Chromatography buffer preparation

All oligo-dT affinity chromatography buffers were freshly prepared with European Pharmacopoeia grade nuclease-free water, and molecular biology grade reagents. Materials used to prepare buffers include sodium phosphate monobasic solution (Sigma-Aldrich), sodium phosphate dibasic (Sigma-Aldrich), NaCl solution (Sigma-Aldrich), and EDTA solution (Thermo Fisher Scientific) and Gu-HCl solution (Thermo Fisher Scientific). Sodium phosphate solution, pH 7.0, was prepared by mixing sodium phosphate monobasic solution and sodium phosphate dibasic solution with a ratio of 39:61. Buffers used for oligo-dT chromatography include (1) equilibration and binding buffer, 50 mM sodium phosphate buffer, 0.2–1.2 M NaCl or Gu-HCl used as salt, 5 mM EDTA and nuclease-free water, pH 7.0; (2) elution buffer, nuclease-free water, pH 7.0; and (3) CIP buffer, 0.5 M NaOH.

Batch chromatography method for frontal optimization

The optimization of loading parameters was first performed in batch. As illustrated in Figure S2A, A one-column load-elute method was designed and used on the ÄKTA PCC system (Cytiva) to perform the frontal experiments in batch with the CIMmultus Oligo dT18 1 mL monolithic column (Sartorius) at room temperature. This method started from an equilibration phase, washing the column with equilibration buffer at the flow rate of 5 mL/min for 20 mL. The IVT mixture was diluted with the binding buffer to 0.7 mg/mL and 15 mL of the diluted load sample, including 5 mL for pump wash, was loaded into the column for each load-elute run at the flow rate of 2 mL/min. The column was overloaded with approximately 6 mg of mRNA per milliliter of the column resin to determine the maximum binding capacity. The load phase was followed by a wash phase pumping equilibration buffer into the column at 5 mL/min for 20 mL, then the column was eluted with 10 mL elution buffer at 2 mL/min flow rate. Finally, the column was washed with 20 mL of CIP buffer at 5 mL/min flow rate.

Continuous chromatography method

An ÄKTA PCC system composed of three sets of binary pumps with multiple inlets and outlets, a UV sample detector (280 nm), a breakthrough UV detector (280 nm), a flow-through UV-vis detector (280 nm), a multi-wavelength elution UV detector (set at 280 nm), a conductometer and a pH monitor were utilized for continuous chromatography. UNICORN Version 7.8 (Cytiva) software was used for method editing, system control and data acquisition. Four 1-mL CIMmultus Oligo dT18 1 mL monolithic columns were used to perform the continuous chromatography experiments.

In the multi-column continuous chromatography system, multiple operation phases take place on different columns simultaneously to ensure the continuity of the chromatography process. The continuous chromatography method starts from an equilibration and start-up phase when all four columns are washed simultaneously with 20 mL of equilibration buffer per column at the flow rate of 5 mL/min. The loop then starts as the schematic in Figure S2B shows, while column one is loaded at 4 mL/min for 20 mL, column two is connected in series to column one to catch the breakthrough from column one. Simultaneously, column three is undertaking regeneration with CIP buffer and equilibration at 10 mL/min for 25 mL and 20 mL, respectively, and column four is performing wash and elution at 10 mL/min for 30 mL and 10 mL, respectively. When the loading to column one is completed, columns switch to wash the weakly bonded mRNA from column one to column three at 10 mL/min for 10 mL. Meanwhile, column two is loading independently, and column four performed regeneration. The columns then switch again to load column two to column three and other columns are performing different phases in sequence continuously as programmed. The close-down phase is conducted when the desired number of loops are completed, washing all columns with CIP buffer at 5 mL/min for 10 min and then equilibration buffer at 5 mL/min for 10 min.

Analytical methods

The yield KPI was measured using AEX HPLC.[59] Using Figure S3B as an example, the mRNA and NTPs quantification analysis was performed by separating the mRNA sample from the individual NTPs, this can be seen in the chromatogram of the load. Chromeleon

software Version 7.2.10 (Thermo Fisher Scientific) was used to calculate the peak areas and compare them with calibration curves for each of the NTPs and the mRNA, to quantify their concentrations in the sample. AEX HPLC was performed using an UltiMate 3000 HPLC system (Thermo Fisher Scientific) with a DNAPac PA200 (50 mm × 2.1 mm I.D., Thermo Fisher Scientific) column at a temperature of 25°C. The sample was diluted 1 in 10 times in nuclease-free water to a final concentration of 50–100 ng/μL. The AEX column was injected with 5 μL of the sample. After an initial 3 min equilibration step at 100% mobile phase A (10 mM NaOH), a series of linear gradients were performed as follows: 0%–15% B (10 mM NaOH, 2 M NaCl) in 2 min, 15%–55% B in 1 min, 55%–65% B in 1 min, and 65%–100% B in 1 min all at a flow rate of 0.5 mL/min. Following this, a wash step of 100% B was performed before a linear gradient of 100% to 0% B in 0.1 min and a re-equilibration step of 100% A in 0.4 min, again, all at a flow rate of 0.5 mL/min. Detection of nucleic acids and NTPs was carried out using UV absorbance at the wavelength of 260 nm. Moreover, the yield was also measured by the UV absorbance at 260 nm using the NanoDrop One C Microvolume UV-Vis Spectrophotometer (Thermo Fisher Scientific) after silica spin column purification using the Monarch RNA Cleanup Kit 500 μg (New England Biolabs), for initial yield measurement after IVT.

The mRNA purity CQA was also measured simultaneously with yield by AEX-HPLC, as details given above. The Chromeleon software Version 7.2.10 (Thermo Fisher Scientific) was used to calculate the level of NTPs remaining in the analytes and compared them with calibration curves, examples are shown in Figure S3B.

The mRNA integrity CQA was measured by CGE, separating and detecting fragments of mRNA based on their sizes was performed on a 5200 Fragment Analyzer System (Agilent).[66] This method uses the DNF-471 RNA Kit (15 nt) (Agilent), which is composed of RNA separation gel, dsDNA inlet buffer, TE rinse buffer, intercalating dye, RNA diluent marker (15 nt), RNA ladder (from 200 to 6,000 nt), capillary conditioning solution. The capillary cassette used was FA 12-Capillary Array Short, 33 cm (Agilent). For this method, the purified mRNA sample was diluted 1 in 10 in nuclease-free water to a concentration of around 50 ng/μL, then 1 in 12 in RNA diluent marker to a final concentration of around 4 ng/μL. Before each separation, a pre-run voltage was applied (8 kV for 30 s), the capillaries were conditioned with the conditioning solution and the capillaries were dipped twice in the rinse buffer. After this, the capillaries were filled with RNA separation gel (by pressure) and then the sample was introduced using a voltage injection (5 kV for 4 s). The separation was then conducted by applying a voltage of 8 kV for 45 min. Detection was carried out using laser-induced fluorescence, by fluorescent dye tagging of the RNA (intercalating dye in the separation gel). Examples of CGE analysis are shown in Figure S3A. The analytical measurement errors for AEX-HPLC, UV-spectroscopy and CGE are provided in Table S1.

DATA AND CODE AVAILABILITY

This study includes no data deposited in external repositories.

ACKNOWLEDGMENTS

This study was funded by the Wellcome Leap R3 Program. The funder played no role in study design, data collection, analysis and interpretation of data, or the writing of this manuscript. This research was funded in whole, or in part, by the Wellcome Trust. For the purpose of Open Access, the author has applied the CC BY public copyrigth licence to ant Author Accepted Manuscript version arrising from this submission.

AUTHOR CONTRIBUTIONS

Conceptualization: J.Q., A.N., M.M., M.J.D., and Z.K.; methodology: J.Q., E.N.W., M.M, M.J.D., and Z.K.; data acquisition: J.Q., A.N., G.W.M., K.A., Z.Y. E.N., E.N.W., and Z.K.; writing – original draft: J.Q., A.N., G.W.M., and Z.K.; writing – review & editing: J.Q., A.N., M.J.D., and Z.K; resources and supervision: M.J.D. and Z.K.

DECLARATION OF INTERESTS

The authors declare no conflict of interest.

SUPPLEMENTAL INFORMATION

Supplemental information can be found online at https://doi.org/10.1016/j.omtn.2024.102333.

REFERENCES

1. Qin, S., Tang, X., Chen, Y., Chen, K., Fan, N., Xiao, W., Zheng, Q., Li, G., Teng, Y., Wu, M., and Song, X. (2022). mRNA-based therapeutics: powerful and versatile tools to combat diseases. Signal Transduct. Targeted Ther. *7*, 166–235. https://doi.org/10.1038/s41392-022-01007-w.
2. Hogan, M.J., and Pardi, N. (2022). mRNA Vaccines in the COVID-19 Pandemic and Beyond. Annu. Rev. Med. *73*, 17–39. https://doi.org/10.1146/ANNUREV-MED-042420-112725.
3. Whitley, J., Zwolinski, C., Denis, C., Maughan, M., Hayles, L., Clarke, D., Snare, M., Liao, H., Chiou, S., Marmura, T., et al. (2022). Development of mRNA manufacturing for vaccines and therapeutics: mRNA platform requirements and development of a scalable production process to support early phase clinical trials. Transl. Res. *242*, 38–55. https://doi.org/10.1016/J.TRSL.2021.11.009.
4. Rosa, S.S., Prazeres, D.M.F., Azevedo, A.M., and Marques, M.P.C. (2021). mRNA vaccines manufacturing: Challenges and bottlenecks. Vaccine *39*, 2190–2200. https://doi.org/10.1016/J.VACCINE.2021.03.038.
5. Hosangadi, D., Warmbrod, K.L., Martin, E.K., Adalja, A., Cicero, A., Inglesby, T., Watson, C., Watson, M., and Connell, N. (2020). Enabling emergency mass vaccination: Innovations in manufacturing and administration during a pandemic. Vaccine *38*, 4167–4169. https://doi.org/10.1016/J.VACCINE.2020.04.037.
6. Sell, T.K., Gastfriend, D., Watson, M., Watson, C., Richardson, L., Cicero, A., Inglesby, T., and Connell, N. (2021). Building the global vaccine manufacturing capacity needed to respond to pandemics. Vaccine *39*, 1667–1669. https://doi.org/10.1016/J.VACCINE.2021.02.017.
7. (2023). Report of the WHO Science Council Potential benefits and limitations of mRNA technology for vaccine research and development for infectious diseases and virus-induced cancers.
8. Vetter, F.L., Zobel-Roos, S., Mota, J.P.B., Nilsson, B., Schmidt, A., and Strube, J. (2022). Toward Autonomous Production of mRNA-Therapeutics in the Light of Advanced Process Control and Traditional Control Strategies for Chromatography. Processes *10*, 1868. https://doi.org/10.3390/PR10091868.
9. Mencin, N., Štepec, D., Margon, A., Vidič, J., Dolenc, D., Simčič, T., Rotar, S., Sekirnik, R., Štrancar, A., and Černigoj, U. (2023). Development and scale-up of oligo-dT monolithic chromatographic column for mRNA capture through understanding of base-pairing interactions. Sep. Purif. Technol. *304*, 122320. https://doi.org/10.1016/J.SEPPUR.2022.122320.
10. Svoboda, M., Frost, H.R., and Bosco, G. (2022). Internal oligo(dT) priming introduces systematic bias in bulk and single-cell RNA sequencing count data. NAR Genom. Bioinform. *4*, lqac035. https://doi.org/10.1093/nargab/lqac035.
11. Dewar, E.A., Guterstam, P., Holland, D., Lindman, S., Lundbäck, P., Brito dos Santos, S., Wang, S.C., and Swartz, A.R. (2024). Improved mRNA affinity chromatography binding capacity and throughput using an oligo-dT immobilized electrospun

polymer nanofiber adsorbent. J. Chromatogr. A *1717*, 464670. https://doi.org/10.1016/J.CHROMA.2024.464670.

12. Korenč, M., Mencin, N., Puc, J., Skok, J., Nemec, K.Š., Celjar, A.M., Gagnon, P., Štrancar, A., and Sekirnik, R. (2021). Chromatographic purification with CIMmultus™ Oligo dT increases mRNA stability. Cell Gene Ther. Insights *7*, 1207–1216. https://doi.org/10.18609/cgti.2021.161.
13. Černigoj, U., Vidic, U., Nemec, B., Gašperšič, J., Vidič, J., Lendero Krajnc, N., Štrancar, A., and Podgornik, A. (2016). Characterization of methacrylate chromatographic monoliths bearing affinity ligands. J. Chromatogr. A *1464*, 72–78. https://doi.org/10.1016/j.chroma.2016.08.014.
14. Piao, X., Yadav, V., Wang, E., Chang, W., Tau, L., Lindenmuth, B.E., and Wang, S.X. (2022). Double-stranded RNA reduction by chaotropic agents during *in vitro* transcription of messenger RNA. Mol. Ther. Nucleic Acids *29*, 618–624. https://doi.org/10.1016/j.omtn.2022.08.001.
15. Konstantinov, K.B., and Cooney, C.L. (2015). White Paper on Continuous Bioprocessing May 20-21, 2014 Continuous Manufacturing Symposium (John Wiley and Sons Inc.). https://doi.org/10.1002/jps.24268.
16. Chiang, M.J., Pagkaliwangan, M., Lute, S., Bolton, G., Brorson, K., and Schofield, M. (2019). Validation and optimization of viral clearance in a downstream continuous chromatography setting. Biotechnol. Bioeng. *116*, 2292–2302. https://doi.org/10.1002/bit.27023.
17. Lee, K.-S., Chen, S.-L., Lin, C.-Y., and Chang, J.-S. (2021). Converting waste molasses liquor into biohydrogen via dark fermentation using a continuous bioreactor. Int. J. Hydrogen Energy *46*, 16546–16554. https://doi.org/10.1016/j.ijhydene.2021.02.101.
18. Hubau, A., Minier, M., Chagnes, A., Joulian, C., Silvente, C., and Guezennec, A.G. (2020). Recovery of metals in a double-stage continuous bioreactor for acidic bioleaching of printed circuit boards (PCBs). Sep. Purif. Technol. *238*, 116481. https://doi.org/10.1016/J.SEPPUR.2019.116481.
19. Maria, G. (2020). molecules Model-Based Optimization of a Fed-Batch Bioreactor for mAb Production Using a Hybridoma Cell Culture. Molecules *25*, 5648. https://doi.org/10.3390/molecules25235648.
20. Tiwari, A., Masampally, V.S., Agarwal, A., and Rathore, A.S. (2023). Digital twin of a continuous chromatography process for mAb purification: Design and model-based control. Biotechnol. Bioeng. *120*, 748–766. https://doi.org/10.1002/bit.28307.
21. Schwarz, H., Gomis-Fons, J., Isaksson, M., Scheffel, J., Andersson, N., Andersson, A., Castan, A., Solbrand, A., Hober, S., Nilsson, B., and Chotteau, V. (2022). Integrated continuous biomanufacturing on pilot scale for acid-sensitive monoclonal antibodies. Biotechnol. Bioeng. *119*, 2152–2166. https://doi.org/10.1002/bit.28120.
22. Scheffel, J., Isaksson, M., Gomis-Fons, J., Schwarz, H., Andersson, N., Norén, B., Solbrand, A., Chotteau, V., Hober, S., and Nilsson, B. (2022). Design of an integrated continuous downstream process for acid-sensitive monoclonal antibodies based on a calcium-dependent Protein A ligand. J. Chromatogr. A *1664*, 462806. https://doi.org/10.1016/j.chroma.2022.462806.
23. Coolbaugh, M.J., Varner, C.T., Vetter, T.A., Davenport, E.K., Bouchard, B., Fiadeiro, M., Tugcu, N., Walther, J., Patil, R., and Brower, K. (2021). Pilot-scale demonstration of an end-to-end integrated and continuous biomanufacturing process. Biotechnol. Bioeng. *118*, 3287–3301. https://doi.org/10.1002/bit.27670.
24. Mahajan, E., George, A., and Wolk, B. (2012). Improving affinity chromatography resin efficiency using semi-continuous chromatography. J. Chromatogr. A *1227*, 154–162. https://doi.org/10.1016/j.chroma.2011.12.106.
25. Fischer, L.M., Wolff, M.W., and Reichl, U. (2018). Purification of cell culture-derived influenza A virus via continuous anion exchange chromatography on monoliths. Vaccine *36*, 3153–3160. https://doi.org/10.1016/j.vaccine.2017.06.086.
26. Mendes, J.P., Bergman, M., Solbrand, A., Peixoto, C., Carrondo, M.J.T., and Silva, R.J.S. (2022). Continuous Affinity Purification of Adeno-Associated Virus Using Periodic Counter-Current Chromatography. Pharmaceutics *14*, 1346. https://doi.org/10.3390/pharmaceutics.
27. CMC; VWG (2012). A-VAX: Applying Quality by Design to Vaccines CMC-Vaccines Working Group.
28. Yu, L.X., Amidon, G., Khan, M.A., Hoag, S.W., Polli, J., Raju, G.K., and Woodcock, J. (2014). Understanding pharmaceutical quality by design. AAPS J. *16*, 771–783. https://doi.org/10.1208/S12248-014-9598-3.
29. Grangeia, H.B., Silva, C., Simões, S.P., and Reis, M.S. (2020). Quality by design in pharmaceutical manufacturing: A systematic review of current status, challenges and future perspectives. Eur. J. Pharm. Biopharm. *147*, 19–37. https://doi.org/10.1016/J.EJPB.2019.12.007.
30. Daniel, S., Kis, Z., Kontoravdi, C., and Shah, N. (2022). Quality by Design for Enabling RNA Platform Production Processes (Elsevier Ltd). https://doi.org/10.1016/j.tibtech.2022.03.012.
31. van de Berg, D., Kis, Z., Behmer, C.F., Samnuan, K., Blakney, A.K., Kontoravdi, C., Shattock, R., and Shah, N. (2021). Quality by design modelling to support rapid RNA vaccine production against emerging infectious diseases. NPJ Vaccines *6*, 65. https://doi.org/10.1038/s41541-021-00322-7.
32. Ly, H.H., Daniel, S., Soriano, S.K.V., Kis, Z., and Blakney, A.K. (2022). Optimization of Lipid Nanoparticles for saRNA Expression and Cellular Activation Using a Design-of-Experiment Approach. Mol. Pharm. *19*, 1892–1905. https://doi.org/10.1021/ACS.MOLPHARMACEUT.2C00032.
33. Cui, T., Fakhfakh, K., Turney, H., Güler-Gane, G., Toloczko, A., Hulley, M., and Turner, R. (2023). Comprehensive studies on building a scalable downstream process for mRNAs to enable mRNA therapeutics. Biotechnol. Prog. *39*, e3301. https://doi.org/10.1002/btpr.3301.
34. Grinsted, J., Liddell, J., Bouleghlimat, E., Kwok, K.Y., Taylor, G., Marques, M.P.C., and Bracewell, D.G. (2022). Purification of therapeutic & prophylactic mRNA by affinity chromatography. Cell Gene Ther. Insights *8*, 335–349. https://doi.org/10.18609/cgti.2022.049.
35. Gomis-Fons, J., Andersson, N., and Nilsson, B. (2020). Optimization study on periodic counter-current chromatography integrated in a monoclonal antibody downstream process. J. Chromatogr. A *1621*, 461055. https://doi.org/10.1016/j.chroma.2020.461055.
36. Kuribayashi, K., Hikata, M., Hiraoka, O., Miyamoto, C., and Furuichi, Y. (1988). A rapid and efficient purification of poly(A)-mRNA by oligo(dT)30-Latex. Nucleic Acids Symp. Ser. 61–64.
37. Nag, K., Sarker, M.E.H., Kumar, S., Khan, H., Chakraborty, S., Islam, M.J., Baray, J.C., Khan, M.R., Mahmud, A., Barman, U., et al. (2022). DoE-derived continuous and robust process for manufacturing of pharmaceutical-grade wide-range LNPs for RNA-vaccine/drug delivery. Sci. Rep. *12*, 9394. https://doi.org/10.1038/s41598-022-12100-z.
38. Feng, Y.J., You, X.J., Ding, J.H., Zhang, Y.F., Yuan, B.F., and Feng, Y.Q. (2022). Identification of Inosine and 2′-O-Methylinosine Modifications in Yeast Messenger RNA by Liquid Chromatography-Tandem Mass Spectrometry Analysis. Anal. Chem. *94*, 4747–4755. https://doi.org/10.1021/acs.analchem.1c05292.
39. Baiersdörfer, M., Boros, G., Muramatsu, H., Mahiny, A., Vlatkovic, I., Sahin, U., and Karikó, K. (2019). A Facile Method for the Removal of dsRNA Contaminant from In Vitro-Transcribed mRNA. Mol. Ther. Nucleic Acids *15*, 26–35. https://doi.org/10.1016/J.OMTN.2019.02.018.
40. Eskelin, K., Lampi, M., Coustau, C., Imani, J., Kogel, K.-H., and Poranen, M.M. (2022). Analysis and purification of ssRNA and dsRNA molecules using asymmetrical flow field flow fractionation. J. Chromatogr. A *1683*, 463525. https://doi.org/10.1016/j.chroma.2022.463525.
41. Eon-Duval, A., Gumbs, K., and Ellett, C. (2003). Precipitation of RNA impurities with high salt in a plasmid DNA purification process: Use of experimental design to determine reaction conditions. Biotechnol. Bioeng. *83*, 544–553. https://doi.org/10.1002/bit.10704.
42. Cheng, M.Y., Tao, W.B., Yuan, B.F., and Feng, Y.Q. (2021). Methods for isolation of messenger RNA from biological samples (Royal Society of Chemistry). https://doi.org/10.1039/d0ay01912g.
43. Girard, V., Hilbold, N.J., Ng, C.K.S., Pegon, L., Chahim, W., Rousset, F., and Monchois, V. (2015). Large-scale monoclonal antibody purification by continuous chromatography, from process design to scale-up. J. Biotechnol. *213*, 65–73. https://doi.org/10.1016/J.JBIOTEC.2015.04.026.
44. Yao, K., Yun, J., Shen, S., Wang, L., He, X., and Yu, X. (2006). Characterization of a novel continuous supermacroporous monolithic cryogel embedded with nanoparticles for protein chromatography. J. Chromatogr. A *1109*, 103–110. https://doi.org/10.1016/j.chroma.2006.01.014.

45. Steinebach, F., Müller-Späth, T., and Morbidelli, M. (2016). Continuous Countercurrent Chromatography for Capture and Polishing Steps in Biopharmaceutical Production (Wiley-VCH Verlag). https://doi.org/10.1002/biot.201500354.

46. Ng, C.K., Rousset, F., Valery, E., Bracewell, D.G., and Sorensen, E. (2014). Design of high productivity sequential multi-column chromatography for antibody capture. Food Bioprod. Process. *92*, 233–241. https://doi.org/10.1016/J.FBP.2013.10.003.

47. Tugcu, N., Roush, D.J., and Göklen, K.E. (2008). Maximizing productivity of chromatography steps for purification of monoclonal antibodies. Biotechnol. Bioeng. *99*, 599–613. https://doi.org/10.1002/BIT.21604.

48. Gielen, J., Aviv, H., and Leder, P. (1974). Characteristics of Rabbit Globin mRNA Purification by Oligo(dT) Cellulose Chromatography. Arch. Biochem. Biophys. *163*, 146–154.

49. Green, M.R., and Sambrook, J. (2019). Isolation of Poly(A)+ Messenger RNA Using Magnetic Oligo(dT) Beads. Cold Spring Harb. Protoc. *2019*, 711–714. https://doi.org/10.1101/PDB.PROT101733.

50. Pemberton, R.E., Liberti, P., and Baglioni, C. (1975). Isolation of Messenger RNA from Polysomes by Chromatography on Oligo(dT)-Cellulose. Anal. Biochem. *66*, 18–28.

51. Miklavčič, R., Megušar, P., Kodermac, Š.M., Bakalar, B., Dolenc, D., Sekirnik, R., Štrancar, A., and Černigoj, U. (2023). High Recovery Chromatographic Purification of mRNA at Room Temperature and Neutral pH. Int. J. Mol. Sci. *24*, 14267. https://doi.org/10.3390/IJMS241814267/S1.

52. Kozarski, M., and Dillen Figure, S. (2024). Improving mRNA Quality by Removal of Truncated mRNA Species by Oligodt Purification.

53. Steinebach, F., Ulmer, N., Wolf, M., Decker, L., Schneider, V., Wälchli, R., Karst, D., Souquet, J., and Morbidelli, M. (2017). Design and operation of a continuous integrated monoclonal antibody production process. Biotechnol. Prog. *33*, 1303–1313. https://doi.org/10.1002/BTPR.2522.

54. Zydney, A.L. (2016). Continuous downstream processing for high value biological products: A Review. Biotechnol. Bioeng. *113*, 465–475. https://doi.org/10.1002/BIT.25695.

55. Silva, R.J.S., Mendes, J.P., Carrondo, M.J.T., Marques, P.M., and Peixoto, C. (2020). Continuous chromatography purification of virus-based biopharmaceuticals: A shortcut design method. Methods Mol. Biol. *2095*, 367–384. https://doi.org/10.1007/978-1-0716-0191-4_21.

56. Khanal, O., and Lenhoff, A.M. (2021). Developments and opportunities in continuous biopharmaceutical manufacturing. mAbs *13*, 1903664. https://doi.org/10.1080/19420862.2021.1903664.

57. Müller-Späth, T. (2023). Continuous Countercurrent Chromatography in Protein Purification. Methods Mol. Biol. *2699*, 31–50. https://doi.org/10.1007/978-1-0716-3362-5_3.

58. Rathore, A.S., Agarwal, H., Sharma, A.K., Pathak, M., and Muthukumar, S. (2015). Continuous Processing for Production of Biopharmaceuticals. Prep. Biochem. Biotechnol. *45*, 836–849. https://doi.org/10.1080/10826068.2014.985834.

59. Welbourne, E.N., Loveday, K.A., Nair, A., Nourafkan, E., Qu, J., Cook, K., Kis, Z., and Dickman, M.J. (2024). Anion exchange HPLC monitoring of mRNA *in vitro* transcription reactions to support mRNA manufacturing process development. Front. Mol. Biosci. *11*, 1250833. https://doi.org/10.3389/fmolb.2024.1250833.

60. Lenk, R., Kleindienst, W., Szabó, G.T., Baiersdörfer, M., Boros, G., Keller, J.M., Mahiny, A.J., and Vlatkovic, I. (2024). Understanding the impact of *in vitro* transcription byproducts and contaminants. Front. Mol. Biosci. *11*, 1426129. https://doi.org/10.3389/FMOLB.2024.1426129.

61. Sahin, U., Karikó, K., and Türeci, Ö. (2014). mRNA-based therapeutics — developing a new class of drugs. Nat. Rev. Drug Discov. *13*, 759–780. https://doi.org/10.1038/nrd4278.

62. Karikó, K., Muramatsu, H., Ludwig, J., and Weissman, D. (2011). Generating the optimal mRNA for therapy: HPLC purification eliminates immune activation and improves translation of nucleoside-modified, protein-encoding mRNA. Nucleic Acids Res. *39*, e142. https://doi.org/10.1093/NAR/GKR695.

63. Popova, P.G., Lagace, M.A., Tang, G., and Blakney, A.K. (2024). Effect of *in vitro* transcription conditions on yield of high quality messenger and self-amplifying RNA. Eur. J. Pharm. Biopharm. *198*, 114247. https://doi.org/10.1016/J.EJPB.2024.114247.

64. Pregeljc, D., Skok, J., Vodopivec, T., Mencin, N., Krušič, A., Ličen, J., Nemec, K.Š., Štrancar, A., and Sekirnik, R. (2023). Increasing yield of *in vitro* transcription reaction with at-line high pressure liquid chromatography monitoring. Biotechnol. Bioeng. *120*, 737–747. https://doi.org/10.1002/BIT.28299.

65. Fisher Scientific, T. (2023). NanoDrop One Microvolume UV-Vis Spectrophotometers.

66. (2024). Library QC in NGS workflow, 5200 Fragment Analyzer (Agilent). https://www.agilent.com/en/product/automated-electrophoresis/fragment-analyzer-systems/fragment-analyzer-systems/5200-fragment-analyzer-system-365720#literature.

OMTN, Volume *35*

Supplemental information

Quality by design for mRNA platform purification based on continuous oligo-dT chromatography

Jixin Qu, Adithya Nair, George W. Muir, Kate A. Loveday, Zidi Yang, Ehsan Nourafkan, Emma N. Welbourne, Mabrouka Maamra, Mark J. Dickman, and Zoltán Kis

ANOVA analysis

The three-factor at three-level Box-Behnken factorial design was applied for the ANOVA analysis with the following equation, where X is the independent variable chosen by the experimental design, Y is the process response (dependent variable), k is the number of patterns, i and j are index number of patterns, b_0 is the intercept term, b_i is the linear main effect, b_{ii} is the quadratic effect, b_{ij} is the interaction effect and ε is the random error [31].

S1 $$Y = b_0 + \sum_{i=1}^{k} b_i X_i + \sum_{i=1}^{k} b_{ii} X_i^2 + \sum_{i=1}^{k}\sum_{j>1}^{k} b_{ij} X_i X_j + \varepsilon$$

Productivity calculations

Productivity is defined as the amount of mRNA that can be purified per unit time (e.g. minute) per unit scale of the process (e.g. mL of the chromatography resin). The productivity difference between batch and continuous chromatography was calculated based on two different methods:

1) based on one single run;
2) based on running the chromatography unit operation non-stop for 24 hours.

For the productivity of one single run, the amount of mRNA purified by batch and continuous chromatography per run was 4.26 and 18.47 mg, respectively. The time consumed, excluding start-up equilibration and shut-down clean-in-place (CIP), was 14 minutes for batch and 20 minutes for continuous chromatography. The chromatography resin applied is 1 mL for both (for continuous chromatography, only 1 mL of the resin was fully utilised at a time). Thus, the productivity was calculated as 0.30 mg/min/mL for the batch and 0.92 mg/min/mL for the continuous chromatography unit operations. Therefore, based on the productivity of one single run, continuous chromatography is 3-fold more productive than the batch chromatography unit operation.

For the productivity of running the chromatography system for 24 hours, 5 minutes of preparation time was included for both batch and continuous between every two runs and the time consumed in total, 22 minutes for batch and 25 minutes for continuous, were taken into account, including start-up equilibration and shut-down CIP. For batch chromatography, there was a 5-minute preparation time between consecutive runs, 4.26 mg of mRNA could be purified each cycle, and 1 mL chromatography resin was applied. It was concluded that 53.33 cycles can be conducted in 24 hours, and this has given a productivity of 0.16 mg/min/mL. For continuous chromatography, only one start-up equilibration (5 minutes) was applied in the initial cycle, and 5 minutes of preparation time was included at the beginning, then the system ran non-stop for 24 hours. Each cycle purified 18.47 mg of mRNA, and this concluded with

71.5 cycles (4 bind-elute stages per cycle) to be conducted in 24 hours and given 0.92 mg/min/mL of productivity. Therefore, based on the 24-hour productivity calculation, continuous chromatography is 5.75-fold more productive than the batch chromatography unit operation. These productivity differences are expected to be maintained under Good Manufacturing Practice (GMP) compliant manufacturing conditions as well, where column volumes might be larger, and more time is required to set up the process (e.g. due to documentation, verification and compliance requirements).

Cost calculations

The cost calculation details are available below in Supplementary Table 10. The operating cost distribution for chromatography unit operations in a GMP facility was estimated based on techno-economic modelling results for the mRNA manufacturing process in a GMP facility, and the following distribution of the cost components was assumed [1,2]:

A) raw materials costs 60% of total costs;

B) chromatography resin costs: 30% of total costs;

C) labour costs: 4.5% of total costs;

D) facility and other fixed costs: 5.5% of total costs.

The cost calculation for the raw materials is based on the percentage mRNA yield of the batch and continuous chromatography unit operations, 85% and 92.6%, respectively. This 7.6% (92.6% minus 85%) yield difference accounts for 60% of the total cost savings. Therefore, a 4.5% (7.6% multiplied by 60%) total cost reduction for raw materials is achievable when transitioning from batch to continuous chromatography.

The cost of the chromatography resin is calculated based on the amount of mRNA that can be purified in batch and continuous, using 1 mL of the chromatography resin for bind-elute cycle, 4.26 mg and 4.62 mg, respectively. These numbers were converted from mg to percentage values. Therefore, there is an 8.45% difference between batch and continuous chromatography. The chromatography resin costs account for 30% of the total costs. Therefore, 2.34% (8.45% multiplied by 30%) of the total costs are reduced due to chromatography resin savings when transitioning from batch to continuous chromatography.

The labour cost difference between batch and continuous chromatography was calculated based on the difference in the productivity between batch and continuous chromatography unit operations. The continuous chromatography was more productive than the batch chromatography by a factor of 5.75 over a period of 24 hours. It was assumed that the labour cost in batch chromatography was 100%, which was reduced by the 5.75 productivity factor of

17.39% in the case of continuous chromatography, resulting in a labour cost reduction of 82.61%. Labour costs only account for 4.5% of the total GMP costs of the chromatography unit operation. Therefore, the total labour cost reduction of continuous chromatography compared to batch is 82.61% multiplied by 4.5% equals 3.72%.

The facility and other fixed costs difference between batch and continuous chromatography were calculated based on the difference in the productivity between batch and continuous chromatography unit operations. The continuous chromatography was more productive than the batch chromatography by a factor of 5.75 over a period of 24 hours. It was assumed that the facility and other fixed costs in batch chromatography were 100%, and this was reduced by the 5.75 productivity factor to 17.39% in the case of continuous chromatography, resulting in a facility and other fixed costs reduction of 82.61%. Facility and other fixed costs only account for 5.5% of the total GMP costs of the chromatography unit operation. Therefore, the total labour cost reduction of continuous chromatography compared to batch is 82.61% multiplied by 5.5% equals 4.54%.

The total operating cost reduction when operating a continuous process compared to a batch process is calculated by summing up the cost reduction of all the cost components: 4.56% plus 2.54% plus 3.72 plus 4.54 equals 15.36%.

Cell transfection results

The cell transfection work was performed with human embryonic kidney 293 cells (HEK 293) obtained from the American Type Culture Collection (ATCC, VA, USA). Cells were seeded at a concentration of 3 x 10^5 cells/mL in the medium of FluoroBrite DMEM (Thermo Fisher Scientific, MA, USA), supplemented with 10% fetal bovine serum (FBS, Sigma Aldrich, MO, USA) and 1% penicillin-streptomycin (Gibco, MA, USA). mRNA transfection was performed using the TransIT-mRNA Transfection Kit (Mirusbio, WI, USA) according to the manufacturer's instructions. As shown in Figure S1, 6 different conditions with the same loading concentration of 1000 ng/µL, mRNA purified by batch chromatography and continuous chromatography were transfected in cells, together with crude IVT mRNA, mRNA purified by Monarch purification kit (New England Biolabs, MA, USA), DNA template and cell only as comparison. The detection of the transfected cells was performed using a SpectraMax iD 5 Multi-Mode Microplate Reader (Molecular Devices, CA, USA). Fluorescence was measured with an excitation wavelength of 482 nm and an emission wavelength of 535 nm. The experiment was repeated three times independently; consistent

results were observed across all replications, demonstrating the reliability of the assay conditions.

Table S1. The errors of UV-spectroscopy, Capillary Gel Electrophoresis (CGE) and Anion Exchange High-Performance Liquid Chromatography (AEX HPLC) analytical methods.

Analytical method	Relative standard deviation [%]	Limit of detection [ng]
UV-spectroscopy	2	2
CGE	1.2	1
AEX-HPLC	4	8 for eGFP mRNA detection
		26 for eGFP mRNA quantification

Table S2. Uncertainty and impact score for the risk assessment of the continuous chromatography process, adopted from Simon et al. (2022). [3]

Uncertainty score		Rationale
5	Very high	No information found
4	High	Limited supportive information
3	Moderate	Product understanding
2	Low	Specific non-clinic studies or clinic studies of similar technologies
1	Very low	Clinical studies

Impact score	Rationale
2	No impact
8	Moderate impact
25	Major impact

Table S3. QA assessment criteria for the criticality of the continuous chromatography QAs, the framework was adopted from Simon et al. (2022). [3]

QA
QA/CQA
pCQA
CQA

Impact \ Uncertainty	1	2	3	4	5
2	2	4	6	8	10
8	8	16	24	32	40
25	25	50	75	100	125

Table S4. Risk assessment of quality attributes for the chromatography process, modified from Simon et al. (2022). [3]

CQA	Impact Score - Safety	Impact Score Efficacy	Uncertainty Score - Safety	Uncertainty Score - Efficacy	Severity Score - Safety	Severity Score - Efficacy	Max Severity	Classification	References
**RNA content**	2	8	3	3	8	24	24	CQA*	4
**RNA sequence identity**	8	25	4	3	32	75	75	CQA	4
**RNA structure integrity**	8	25	4	3	32	75	75	CQA	4
**5' capping efficiency**	2	25	3	2	6	50	50	CQA	5–7
**PolyA tail length**	2	25	4	3	8	75	75	CQA	6,8
**PolyA tail level**	8	25	4	2	32	50	50	CQA	6,8
**RNA purity**	25	25	2	3	50	75	75	CQA	9
**dsRNA species**	25	25	2	3	50	75	75	CQA	10,11
**Shorter RNA species**	25	25	3	3	75	75	75	CQA	12
**RNA precipitates**	2	8	4	4	8	32	32	pCQA	4
**Residual DNA template**	8	8	3	3	24	24	24	QA	13
**Residual host cell proteins**	8	8	4	4	32	32	32	pCQA	14,15
**Residual enzymes**	8	25	4	3	32	75	75	pCQA	16,17
**Residual impurities**	8	8	3	4	24	32	32	pCQA	18,19
**Residual solvents**	8	8	3	4	24	32	32	pCQA	20
**pH**	2	25	3	3	6	75	75	CQA	12,21
**Appearance**	2	2	3	3	6	6	6	QA	22
**Viscosity**	2	2	4	3	8	6	8	QA	23
**Immunogenicity**	8	25	2	3	50	24	50	CQA	5,24–27
**Potency / In Vitro Expression**	2	25	2	2	50	4	50	CQA	12,28–30

Table S5. Assessment criteria for evaluating the uncertainty and impact of the CPPs and CMAs on product CQAs and manufacturing KPIs.

Uncertainty assessment		Impact assessment	
Uncertainty rating	Uncertainty rating rationale	Impact score*	Descriptor
High^4	Limited supportive information about the relation.	0	Negligible
Moderate^3	Process expert knowledge only, or information taken from other similar processes.	+, -, ±,∓1	Minor
Low^2	Relation is supported by both expert knowledge and the literature on the same or very similar processes.	+, -, ±,∓2	Moderate
Very Low^1	Relation is supported by a large number and consistent published data or results, information specific to the mRNA process.	+, -, ±,∓3	Major

* The "^4 to ^1" in superscript in the table indicates the significance of uncertainty, from very high uncertainty "^1" to very low uncertainty "^1". The "+" indicates a positive linear slope, the "-" sign indicates a negative linear slope, and the "±" indicates a peak trend (initial increase, reaching a maximum followed by a decrease) when plotting CQAs or KPIs as a function of PPs or CMAs. The "∓" indicates a valley trend (opposite, inverse of the peak trend), an initial decrease, reaching a minimum followed by an increase when plotting CQAs or KPIs as a function of PPs or CMAs.

Table S6. Definitions of Key Performance Indicators (KPIs)

**KPI**	**Definition**
mRNA yield [mg]	The amount of mRNA recovered after the purification by chromatography
Eluted mRNA amount [mg]	The amount of mRNA eluted in the elution phase

Process time [min]	The processing time used for the chromatography process, excluding the time for the start-up equilibration and shut-down phases
Column switch interval [min]	The time measurement between the column switches, as the column switch is dictated by the Breakthrough UV absorbance at 280 nm
Phase time for continuity [min]	The time measurement for each phase time, as the consideration for the continuity of the chromatography process
Productivity [mg/min/mL]	The amount of mRNA purified per minute per mL of the chromatography resin

Table S7. The independent and dependent variables of the experimental optimisation.

Independent variable	Low level (-1)	Medium level (0)	High level (+1)	Dependent variable
X1: guanidine-HCl concentration (M)	0.3	0.6	0.9	Y1 = yield (mg)
X2: mRNA load concentration (mg/mL)	0.3	0.5	0.7	Y2 = mRNA integrity (%)
X3: Load flow rate (mL/min)	1	3	5	Y3 = purity (%)

Table S8. Output of ANOVA analysis.
$Yield=70.304-3.84\times X1-15.107\times X2+7.194\times X3-2.0562\times X1^2+7.9088\times X2^2$
$14.256\times X3^2+1.75\times X1X2+7.535\times X1X3+0.8925\times X2X3$

Factor	Sum of squares	DF	Mean of squares	F	P Value
X1	147.46	1	147.46	2.346	0.17649
X2	2282.2	1	2282.2	36.31	0.00094307
X3	517.54	1	517.54	8.234	0.028446
$X1^2$	11.147	1	11.147	0.17734	0.68834
$X2^2$	164.9	1	164.9	2.6236	0.15641
$X3^2$	535.81	1	535.81	8.5248	0.026641
X1X2	24.5	1	24.5	0.3898	0.55593
X1X3	454.21	1	454.21	7.2265	0.096139
X2X3	6.3724	1	6.3724	0.10139	0.76096
Residual error	377.12	6	52.854		
Total	4473	15	298.2		

Table S9. The independent and dependent variables of the experimental optimisation

Independent variable	Low level (-1)	Medium level (0)	High level (+1)	Dependent variable
X1: mRNA load concentration (mg/mL)	0.1	0.25	0.4	Y1 = yield (mg)
X2: Load flow rate (mL/min)	2	4	6	Y2 = mRNA integrity (%)<br>Y3 = purity (%)

Table S10. Output of ANOVA analysis, Yield=a-b×X1-c×X2+d× $X1^2$+e×$X2^2$-f×X1X2

Factor	Sum of squares	DF	Mean of squares	F	P Value
X1	0.081448	1	0.081448	92.783	0.002375
X2	0.0029007	1	0.0029007	3.3044	0.16669
$X1^2$	0.018348	1	0.018348	20.902	0.019635
$X2^2$	0.0022916	1	0.0022916	2.6106	0.20457
X1X2	0.011618	1	0.011618	13.235	0.035792
Residual error	0.0026335	3	0.00087784		
Total	0.11924	8	0.014905		

Table S11. Details of operating cost calculations for batch vs continuous chromatography.

	**Batch Chromatography**	**Continuous Chromatography**
Amount of mRNA purified each cycle [mg][(A)]	4.26	18.47
Time consumed excluding start-up and shut-down [min][(B)]	14	20
Time consumed in total [min][(C)]	22	25
Chromatography resin applied [mL][(D)]	1	1
Preparation time [min][(E)]	5	5
Productivity [mg/min/mL] for one run[(F)]	0.30	0.92
Productivity [mg/min/mL] of running for 24 hours[(G)]	0.16	0.92

Productivity increase for a single run: 3.07 fold
Productivity increase of running for 24 hours: 5.75 fold

(A) The amount of mRNA recovered from the chromatography process, quantified by AEX HPLC.
(B) The time consumed for load, wash and elution phases, excluding start-up equilibration and shut-down column CIP.
(C) The total process time including start-up equilibration, load, wash, elution and shut-down column CIP.

(D) The chromatography resin that is fully utilised. For continuous chromatography, only 1 mL resin is fully utilised at a time, as the second column in the loading zone is only catching the flowthrough mRNA from the first column.
(E) The time consumed for preparing buffers, samples equipment and consumables.
(F) The productivity calculated for a single run, considering the amount of mRNA purified each cycle, time consumed excluding start-up and shut-down and chromatography resin applied.
(G) The productivity calculated for running the chromatography process for 24 hours non-stop, considering the amount of mRNA purified each cycle, time consumed in total, chromatography resin applied and preparation time.

Table S12. Details of operating cost calculations for batch vs continuous chromatography.

	Batch Chromatography	Continuous Chromatography	Difference [%]	Proportion of total costs [%]*	Cost reduction [%]
Raw materials costs [%] (A)	85	92.6	7.6	60	4.56
Chromatography resin costs [%] (B)	4.26	4.62	8.45	30	2.54
Labour costs [%] (C)	100	17.39	82.61	4.5	3.72
Facility and other costs [%] (D)	100	17.39	82.61	5.5	4.54
				**Total cost reduction:**	**15.36 %**

* The proportion of total operating costs was calculated based on techno-economic modelling results[1,2].
(A) The raw materials cost difference was calculated based on yield differences between batch (85%) and continuous (92.6%) chromatography.
(B) The chromatography resin cost difference was calculated based on the difference in the amount of mRNA purified per mL of resin between batch and continuous chromatography.
(C) The labour cost difference was calculated based on the difference in the productivity between batch and continuous, assuming batch at 100% cost and continuous was reduced by the productivity factor difference (of 5.75) between batch and continuous.
(D) The facility and other costs difference was calculated based on the difference in the productivity between batch and continuous, assuming batch at 100% cost and continuous was reduced by the productivity factor difference (of 5.75) between batch and continuous.

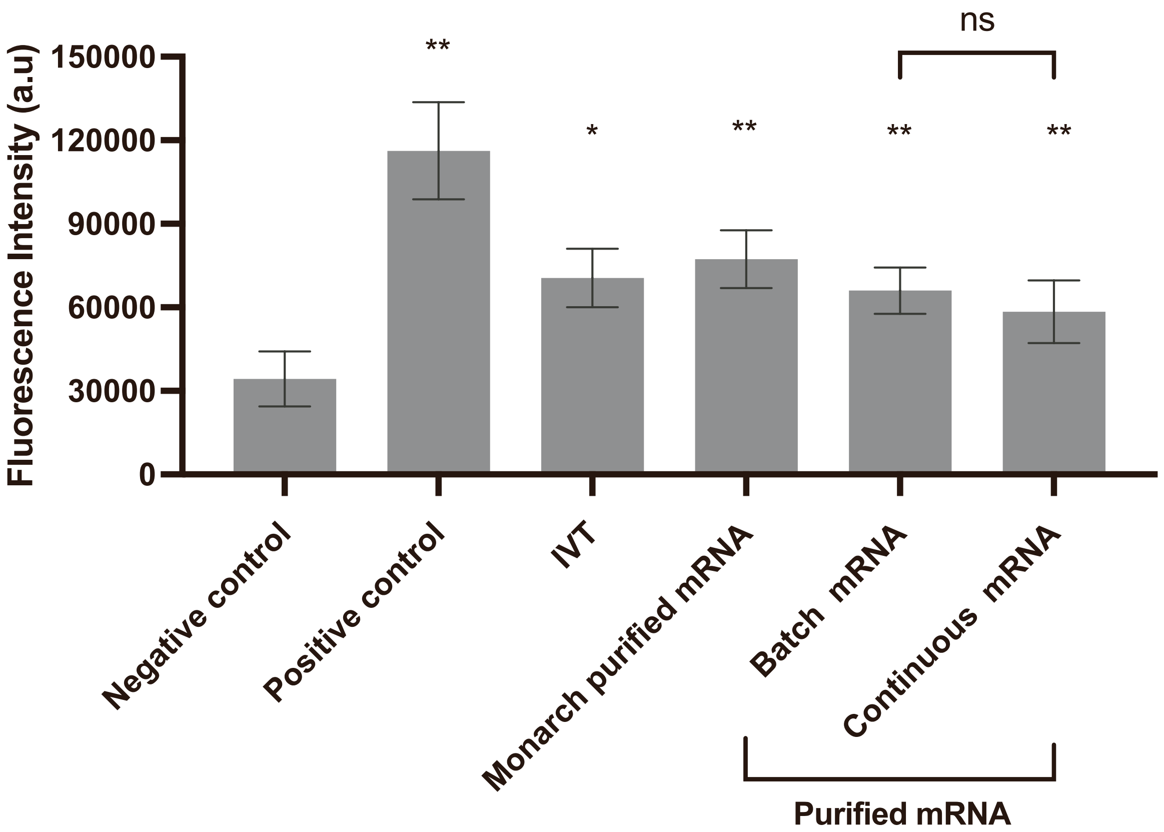

Figure S1. Fluorescence measurements of various mRNA samples using the Spectra Max 24-well plate reader (Excitation: 485 nm, Emission: 535 nm). The samples tested include negative control (cell only), positive control (plasmid DNA), IVT, Monarch kit purified mRNA, mRNA purified by batch chromatography and mRNA purified by continuous chromatography. The results demonstrate the fluorescence intensity of each sample after 24 hours of incubation, indicating the relative expression levels. Statistical significance is indicated as follows: *, $p<0.05$ (significant), **, $p<0.01$ (very significant) and ns (not significant) compared to the negative control.

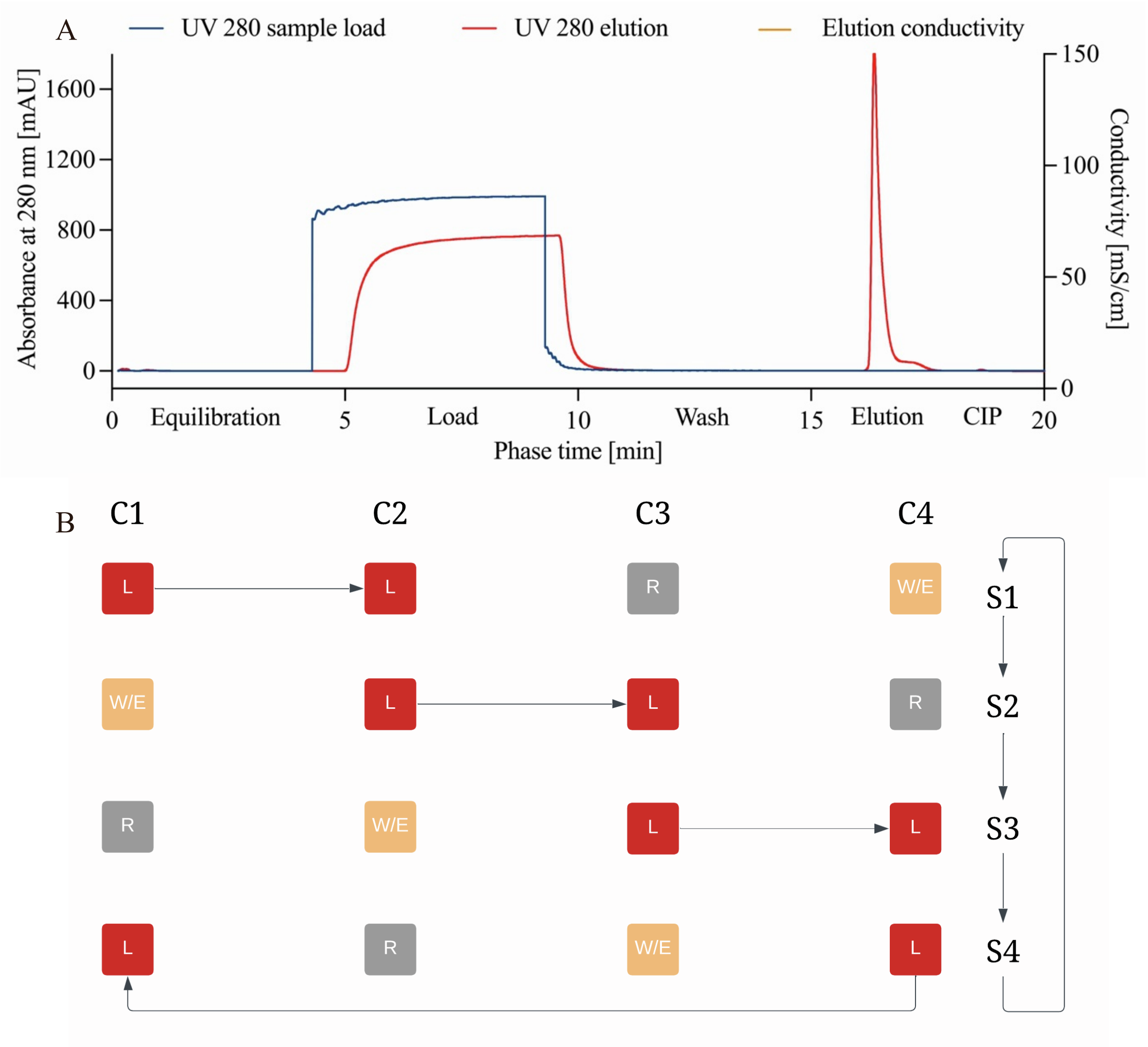

Figure S2. Oligo-dT batch chromatogram and illustration of the continuous chromatography process. **A**: One column in-batch oligo-dT chromatography process. **B.** Schematic of four-column periodic counter-current (PCC) continuous chromatography. C1-C4: columns 1-4; L: Load; W: wash; R: regeneration; E: elution and S: switch.

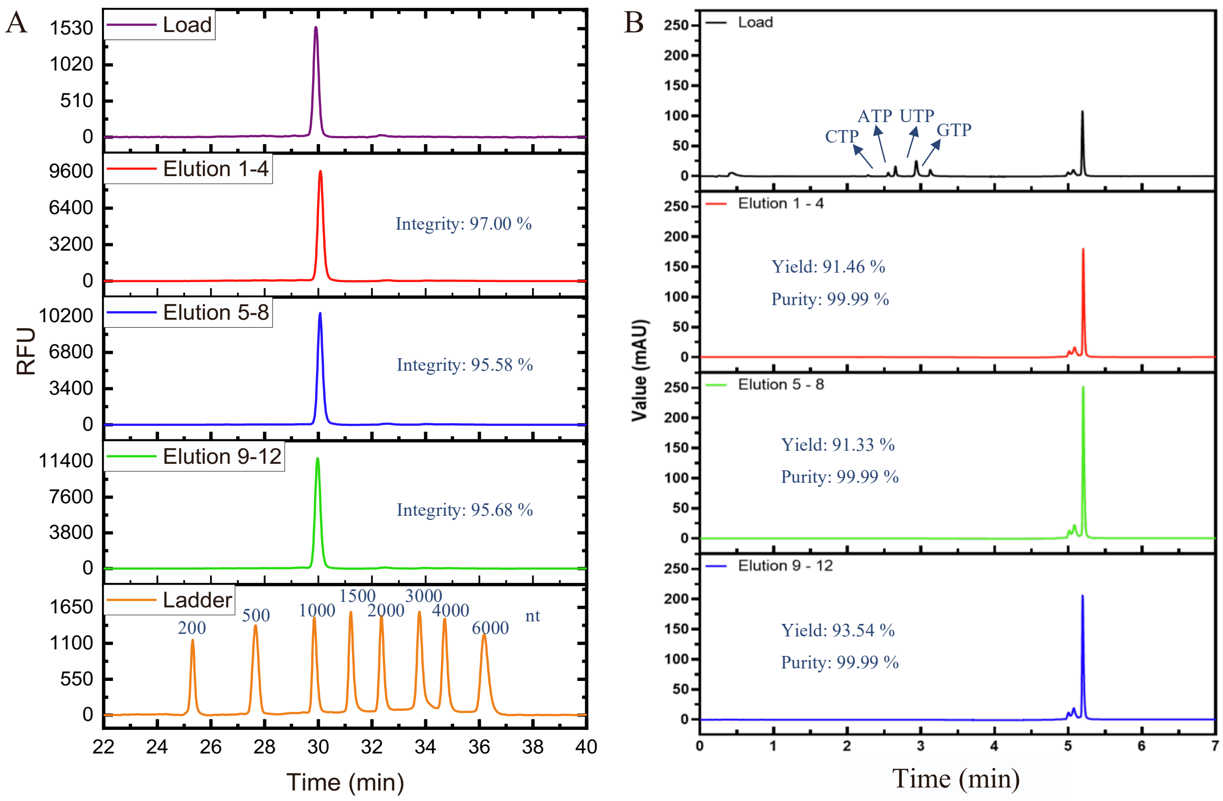

Figure S3. A representative example of CGE and HPLC analysis results. **A**. Examples of CGE analysis of mRNA integrity. mRNA fragments are separated and detected by fluorescence based on their sizes and the integrity of RNA is determined by comparing with the mRNA ladder. Examples of AEX HPLC analysis of mRNA yield and purity. AEX HPLC separates the molecules based on their charge and quantifies the amount of mRNA and NTPs using UV absorbance at the wavelength of 260 nm.

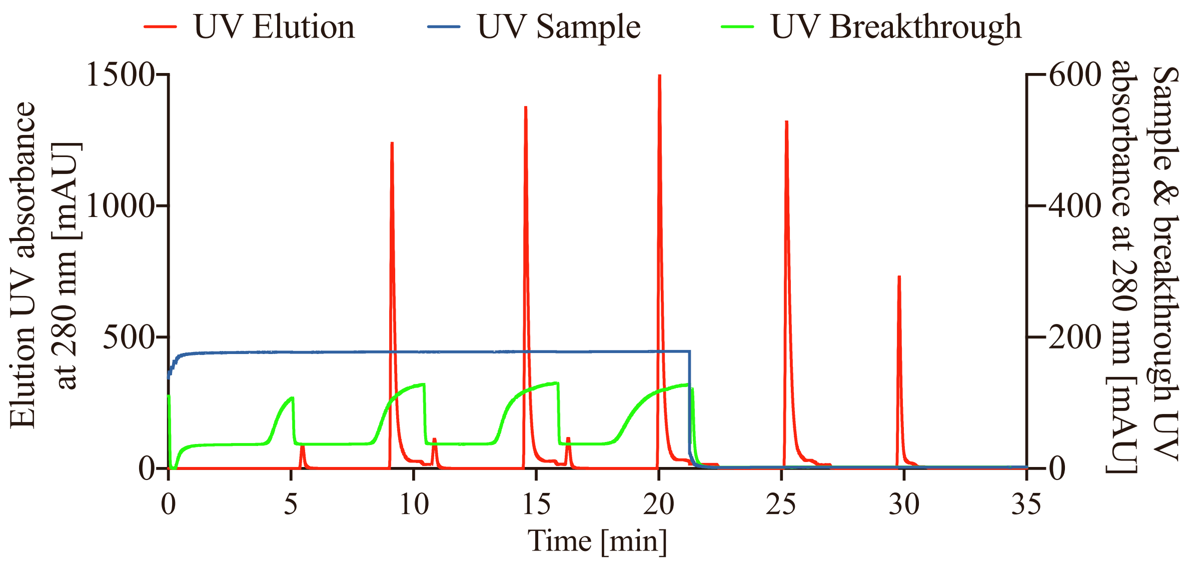

Figure S4. Chromatogram of the continuous oligo-dT purification of the 4284 nucleotides long SARS-CoV-2 Spike Protein encoding mRNA. The total salt concentration was 600 mM guanidine-HCl, the SARS-CoV-2 Spike Protein mRNA load concentration was 0.1 mg/mL, the load volume was 20 mL for each load-elute loop and the load flow rate was 4 mL/min. In total, 1 cycle of 4 load-elute loops was performed using four 1 mL monolith oligo-dT columns under the same conditions as the test performed with eGFP encoding mRNA. The total run time was 32 minutes, excluding the start-up equilibration and shut-down phases. In total, 7.56 mg of purified mRNA was eluted out of the 8 mg unpurified crude IVT mRNA loaded, giving a yield of 94.5%. The purified mRNA integrity was >90% and the purity was 99.9%.

References

1. Kis, Z., Kontoravdi, C., Shattock, R., and Shah, N. (2020). Resources, Production Scales and Time Required for Producing RNA Vaccines for the Global Pandemic Demand. Vaccines 2021, Vol. 9, Page 3 *9*, 3. 10.3390/VACCINES9010003.
2. Kis, Z., Tak, K., Ibrahim, D., Papathanasiou, M.M., Chachuat, B., Shah, N., and Kontoravdi, C. (2022). Pandemic-response adenoviral vector and RNA vaccine manufacturing. npj Vaccines 2022 7:1 *7*, 1–10. 10.1038/s41541-022-00447-3.
3. Daniel, S., Kis, Z., Kontoravdi, C., and Shah, N. (2022). Quality by Design for enabling RNA platform production processes. Preprint at Elsevier Ltd, 10.1016/j.tibtech.2022.03.012 10.1016/j.tibtech.2022.03.012.
4. van de Berg, D., Kis, Z., Behmer, C.F., Samnuan, K., Blakney, A.K., Kontoravdi, C., Shattock, R., and Shah, N. (2021). Quality by design modelling to support rapid RNA vaccine production against emerging infectious diseases. NPJ Vaccines *6*. 10.1038/s41541-021-00322-7.
5. Evaluation of the quality, safety and efficacy of messenger RNA vaccines for the prevention of infectious diseases: regulatory considerations (2021).
6. Chang Kim, S., Singh Sekhon, S., Shin, W.-R., Ahn, G., Cho, B.-K., Ahn, J.-Y., and Kim, Y.-H. (2022). Modifications of mRNA vaccine structural elements for improving mRNA stability and translation efficiency. *18*, 1–8. 10.1007/s13273-021-00171-4.
7. Decroly, E., Ferron, F., Lescar, J., and Canard, B. (2011). Pre-mRNA splicing Conventional and unconventional mechanisms for capping viral mRNA. Nat Rev Microbiol *10*, 51. 10.1038/nrmicro2675.
8. Bernstein, P., and Ross, J. (1989). Quantum Mechanical Tunneling in Biological Systems.) Acc. ('hem. Res *14*, 13650–13662.

9. Vermeulen, J., De Preter, K., Lefever, S., Nuytens, J., De Vloed, F., Derveaux, S., Hellemans, J., Speleman, F., and Vandesompele, J. (2011). Measurable impact of RNA quality on gene expression results from quantitative PCR. Nucleic Acids Res *39*. 10.1093/nar/gkr065.

10. Foster, J.B., Choudhari, N., Perazzelli, J., Storm, J., Hofmann, T.J., Jain, P., Storm, P.B., Pardi, N., Weissman, D., Waanders, A.J., et al. Purification of mRNA Encoding Chimeric Antigen Receptor Is Critical for Generation of a Robust T-Cell Response. 10.1089/hum.2018.145.

11. Karikó, K., Muramatsu, H., Ludwig, J., and Weissman, D. (2011). Generating the optimal mRNA for therapy: HPLC purification eliminates immune activation and improves translation of nucleoside-modified, protein-encoding mRNA. Nucleic Acids Res *39*, e142–e142. 10.1093/NAR/GKR695.

12. CHMP (2021). COVID-19 Vaccine Moderna, INN-COVID-19 mRNA Vaccine (nucleoside modified).

13. Wang, Y., Cooper, R., Kiladjian, A., Bergelson, S., and Feschenko, M. (2019). A Digestion-free Method for Quantification of Residual Host Cell DNA in rAAV Gene Therapy Products. Mol Ther Methods Clin Dev *13*, 526–531. 10.1016/j.omtm.2019.05.005.

14. Pharmacopeia, U.S. (2016). Residual Host Cell Protein Measurement in Biopharmaceuticals USP 39 NF 34 THE NATIONAL FORMULARY á1132ñ Residual Host Cell Protein Measurement in Biopharmaceuticals.

15. Singh Rathore, A., Sobacke, S.E., Kocot, T.J., Morgan, D.R., Dufield, R.L., and Mozier, N.M. (2003). Analysis for residual host cell proteins and DNA in process streams of a recombinant protein product expressed in Escherichia coli cells. J Pharm Biomed Anal *32*, 1199–1211. 10.1016/S0731-7085(03)00157-2.

16. Maruggi, G., Zhang, C., Li, J., Ulmer, J.B., and Yu, D. (2019). mRNA as a Transformative Technology for Vaccine Development to Control Infectious Diseases. Molecular Therapy *27*. 10.1016/j.ymthe.2019.01.020.

17. Tagliabue, A., Stadler, K., Rauch, S., Jasny, E., Schmidt, K.E., and Petsch, B. (2018). New Vaccine Technologies to Combat Outbreak Situations. Frontiers in Immunology | www.frontiersin.org *9*. 10.3389/fimmu.2018.01963.

18. Mathieu, E., Ritchie, H., Ortiz-Ospina, E., Roser, M., Hasell, J., Appel, C., Giattino, C., and Rodés-Guirao, L. (2020). Coronavirus Pandemic (COVID-19). Our World in Data *5*, 947–953. 10.1038/S41562-021-01122-8.

19. Lanigan, R.S., Yamarik, T.A., and Andersen, F.A. (2002). Final report on the safety assessment of EDTA, calcium disodium EDTA, diammonium EDTA, dipotassium EDTA, disodium EDTA, TEA-EDTA, tetrasodium EDTA, tripotassium EDTA, trisodium EDTA, HEDTA, and trisodium HEDTA. Int J Toxicol *21 Suppl 2*, 95–142. 10.1080/10915810290096522.
20. Step ICH guideline Q3C (R8) on impurities: guideline for residual solvents.
21. van der Helm, M.W., van der Meer, A.D., Eijkel, J.C.T., van den Berg, A., and Segerink, L.I. (2016). Microfluidic organ-on-chip technology for blood-brain barrier research. https://doi.org/10.1080/21688370.2016.1142493 *4*. 10.1080/21688370.2016.1142493.
22. Poveda, C., Biter, A.B., Bottazzi, M.E., Strych, U., Biter@bcm, A., and Edu, A.B.B. Establishing Preferred Product Characterization for the Evaluation of RNA Vaccine Antigens. 10.3390/vaccines7040131.
23. EUROPEAN PHARMACOPOEIA.
24. CHMP Committee for Medicinal Products for Human Use (CHMP) Assessment report Comirnaty Common name: COVID-19 mRNA vaccine (nucleoside-modified).
25. Vlatkovic, I. (2021). Non-Immunotherapy Application of LNP-mRNA: Maximizing Efficacy and Safety. Biomedicines 2021, Vol. 9, Page 530 *9*, 530. 10.3390/BIOMEDICINES9050530.
26. Kalnin, K. V, Plitnik, T., Kishko, M., Zhang, J., Zhang, D., Beauvais, A., Anosova, N.G., Tibbitts, T., DiNapoli, J., Ulinski, G., et al. ARTICLE Immunogenicity and efficacy of mRNA COVID-19 vaccine MRT5500 in preclinical animal models. 10.1038/s41541-021-00324-5.
27. Heine, A., Juranek, S., and Brossart, P. (2021). Clinical and immunological effects of mRNA vaccines in malignant diseases. Preprint at BioMed Central Ltd, 10.1186/s12943-021-01339-1 10.1186/s12943-021-01339-1.
28. Corbett, K.S., Flynn, B., Foulds, K.E., Francica, J.R., Boyoglu-Barnum, S., Werner, A.P., Flach, B., O'Connell, S., Bock, K.W., Minai, M., et al. (2020). Evaluation of the mRNA-1273 Vaccine against SARS-CoV-2 in Nonhuman Primates. New England Journal of Medicine *383*, 1544–1555. 10.1056/NEJMOA2024671/SUPPL_FILE/NEJMOA2024671_DISCLOSURES.PDF.
29. Sanyal, G., Särnefält, A., and Kumar, A. (2021). Considerations for bioanalytical characterization and batch release of COVID-19 vaccines. Preprint at Nature Research, 10.1038/s41541-021-00317-4 10.1038/s41541-021-00317-4.

30. Rahman, M.M., Zhou, N., and Huang, J. (2021). An Overview on the Development of mRNA-Based Vaccines and Their Formulation Strategies for Improved Antigen Expression In Vivo. 10.3390/vaccines9030244.

31. Nourafkan, E., Gao, H., Hu, Z., and Wen, D. (2017). Formulation optimization of reverse microemulsions using design of experiments for nanoparticles synthesis. Chemical Engineering Research and Design *125*, 367–384. 10.1016/j.cherd.2017.07.023.
